# Anomalous twin boundaries in two dimensional materials

**DOI:** 10.1038/s41467-018-06074-8

**Published:** 2018-09-05

**Authors:** A. P. Rooney, Z. Li, W. Zhao, A. Gholinia, A. Kozikov, G. Auton, F. Ding, R. V. Gorbachev, R. J. Young, S. J. Haigh

**Affiliations:** 1 School of Materials, University of Manchester, Manchester, M13 9PL UK; 2 National Graphene Institute, University of Manchester, Manchester, M13 9PL UK; 3 Institute of Textiles and Clothing, Polytechnic University, Hung Hom, Hong Kong; 4 Center for Multidimensional Carbon Materials, Institute for Basic Science (IBS-CMCM)/School of Material Science and Engineering, Ulsan National Institute of Science and Technology (UNIST), Ulsan, 44919 Korea; 5 Manchester Centre for Mesoscience and Nanotechnology, University of Manchester, Manchester, M13 9PL UK; 6 School of Physics and Astronomy, University of Manchester, Oxford Road, Manchester, M13 9PL UK

## Abstract

Twin boundary defects form in virtually all crystalline materials as part of their response to applied deformation or thermal stress. For nearly six decades, graphite has been used as a textbook example of twinning with illustrations showing atomically sharp interfaces between parent and twin. Using state-of-the-art high-resolution annular dark-field scanning transmission electron microscopy, we have captured atomic resolution images of graphitic twin boundaries and find that these interfaces are far more complex than previously supposed. Density functional theory calculations confirm that the presence of van der Waals bonding eliminates the requirement for an atomically sharp interface, resulting in long-range bending across multiple unit cells. We show these remarkable structures are common to other van der Waals materials, leading to extraordinary microstructures, Raman-active stacking faults, and sub-surface exfoliation within bulk crystals.

## Abstract (web summary)

All materials subjected to mechanical deformation form low energy interfaces known as twin boundaries. Here, the authors investigate a variety of structural features that form upon bending atomically thin 2D-crystals, and predict distinct classes of post deformation microstructure based on their atomic arrangement, bend angle and flake thickness.

## Introduction

Graphene and related two dimensional (2D) crystals display remarkably high mechanical strength and flexibility^1^. Such good flexibility is key for exploiting 2D materials in many emerging technologies such as wearable electronics, bioelectronics, protective coatings and composites^1^ and recently bending has been suggested as a route to tune electronic transport behaviour^2^. For virtually all crystalline materials, macroscopic deformation is accommodated by the movement of dislocations and through the formation of twinning defects^3^; it is the geometry of the resulting microstructure that largely determines the mechanical and electronic properties. Despite this, the atomic microstructure of 2D materials after mechanical deformation has not been widely investigated: only by understanding these deformed microstructures can the resulting properties be accurately predicted and controlled. Here we show the different structural features that can form as a result of bending in van der Waals (vdW) crystals of 2D materials. We find that twin boundaries, an important class of crystal defect, are delocalised by several nm and not atomically sharp as has been assumed for over half a century^4^. In addition, we demonstrate that different classes of microstructure are present in the deformed material and can be predicted from just the atomic structure, bend angle, and flake thickness. We anticipate that this new knowledge of the deformation structure for 2D materials will provide foundations for tailoring transport behaviour^2^, mechanical properties, liquid-phase^5,6^ and scotch-tape exfoliation^7,8^, and crystal growth.

## Results

### Characterisation of anomalous twin boundaries

Twin defects are identifiable by the presence of a twin boundary: a plane of lattice points where the crystals on either side of the interface possess mirror symmetry^9^. The formation of atomically sharp twin boundaries is ubiquitous in virtually all 3D materials including metals^10–12^ and ceramics^13–15^. Our understanding of twinning in graphite has stemmed from the seminal work on twin structures by Friese and Kelly^4^, nearly six decades ago, and has changed remarkably little since^9,16,17^, although recently there has been significant interest in the electronic transport properties of wrinkles, bends and kinks in graphene^2^.

**Fig. 1:**
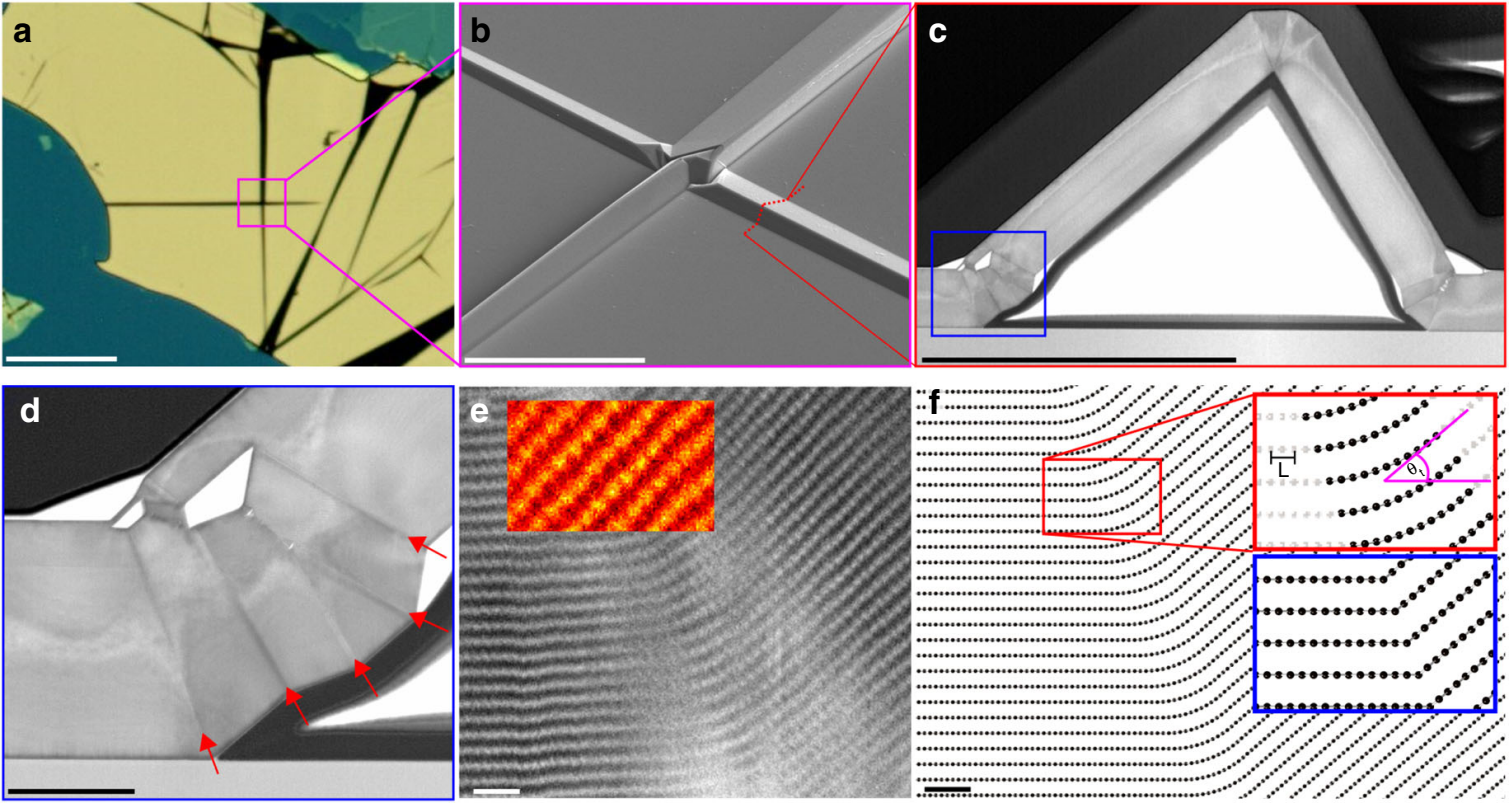
Atomic structure of twin boundaries in layered vdW crystals, **a** Optical plan view image of a 200 nm thick graphite flake on silicon wafer. Stress induced kink bands can be seen intersecting one another at angular multiples of 30° (corresponding to zig-zag, zz, or armchair, ac, crystal directions). Scale bar 100 μm. **b** Scanning electron microscopy (SEM) oblique view of zz kink band meeting an ac kink band at 90°. The site of the FIB cross-section is annotated red. Scale bar 10 μm. **c**, **d** Bright field STEM cross-sectional imaging of the ac kink band. The kink is composed of flat areas of Bernal stacking bordered by many discrete boundaries of almost exactly the same angle, (boundaries annotated with red arrows) which are identified as incommensurate twin boundaries (see SI). Scale bars 1 μm and 100 nm, respectively. **e** Filtered atomic resolution image of a twin boundary in hBN. The inset shows perfect atomic AA’ stacking is maintained either side (parent and twin lattices) of the twin boundary. The core of the boundary is composed of a volume of crystal, which bends like a nanotube and is incommensurately stacked. Scale bar 1 nm. **f** Atomic model schematic comparing the delocalised incommensurate twin boundary structure (main figure and upper panel inset) with the conventional abrupt twin boundary (lower panel inset). The parameters that govern twin geometry, slip length *L* and the ideal twin angle *θ*_*t*_ are annotated (Table 1). Scale bar 1 nm

2D materials are characterised by strong in-plane covalent bonding with weaker vdW bonding between the basal planes. The deformation behaviour of these crystals is therefore highly anisotropic. Slip occurs readily on the basal plane^18^ but is forbidden on prismatic planes as this would require breaking the strong in-plane bonds^4,9^. In contrast, metallic bonding allows many crystallographic slip systems to be active simultaneously, leading to the ductile deformation behaviour of many metals. Twinning in metals occurs to accommodate the strains induced during crystal growth and deformation, with atomic imaging of metallic twin boundaries^11,12^ invariably revealing the atomically sharp crystal interfaces^11,12^ frequently illustrated in material science textbooks (Fig. 1f inset blue box)^3,9^. Twinning in polymers is known to take place by the bending of the molecules across the twin boundary^19^ but no one has previously performed an atomically-detailed analysis of twin boundaries in vdW crystals of 2D materials.

Bending of layered crystals requires neighbouring basal planes to slide past each other, usually as a result of the movement of basal dislocations. It is well known that 2D crystals with hexagonal unit cells, such as graphene, boron nitride and many transition metal dichalcogenides, have preferential low energy edges commonly termed armchair (ac) and zig-zag (zz). Armchair and zig-zag directions are preferred for basal plane dislocation movement and hence serve as preferential directions for folds, bends and kink bands when the material is stressed^20,21^. When basal plane dislocations stack vertically, a twin boundary can appear. This is defined crystallographically as an interface separating two crystals with perfect commensurate stacking and mirror symmetry (see Supplementary Notes 1 and 2). The formation of such boundaries is driven by energy minimisation between the strain energy of the distorted lattice and the vdW energy between adjacent planes (see Supplementary Note 3). To minimise the latter, crystals maintain perfect commensurate layer stacking away from the boundary causing a periodic constraint on the magnitude of the slip between each successive plane. The magnitude of the twin angle, *θ*_*t*_, for an ideal twin boundary is hence simply related to the quantised slip translation vector, **L**_slip_, through the relationship

1 $$\theta _t = 2\,{\rm{tan}}^{ - 1}\left[ {\frac{{{\mathbf{L}}_{{\rm{slip}}}}}{c}} \right],$$

where *c* is the plane-normal lattice vector of the bulk crystal unit cell, equivalent to two basal plane separations for the 2D materials considered in this work (further details in Supplementary Note 1). The predicted values of **L**_slip_ and *θ*_*t*_ for the zz and ac direction twin boundaries in graphite, hBN and MoSe_2_ are listed in Table 1.

**Table 1:** Parameters calculated from literature X-ray crystallography structures for armchair and zig-zag twin boundaries in graphite, hBN and MoSe_2_

	Graphite				hBN		MoSe_2_	
Direction of twin boundary	ac		zz		ac	zz	ac	zz
Slip direction	zz		ac		zz	ac	zz	ac
**L**_slip_	*α*	*α* / √ 3	2*α* / √ 3	3*α* / √ 3	*α*	3*α* / √ 3	*α*	3*α* / √ 3
*c* (nm)	0.67	0.67	0.67	0.67	0.67	0.67	1.23	1.23
a (nm)	0.25	0.25	0.25	0.25	0.25	0.25	0.33	0.33
**L**_slip_ (nm)	0.25	0.14	0.29	0.43	0.25	0.43	0.33	0.56
*θ*_*t*_(°)	40.3	23.9	46.0	64.9	41.2	65.9	30.0	48.6

Higher order twin zz angles in graphite (2*α*/√3 and 3*α*/√3) are likely to be less favourable due to the ease of splitting of such basal dislocations into separate partial dislocations with lower energy

In this paper, we have performed an extensive investigation of atomic morphology of deformation bends in exfoliated vdW materials and have found that twin boundaries are common microstructural features whose presence has previously been largely missed or ignored. In Fig. 1, we present atomic resolution cross-sectional scanning transmission electron microscope (STEM) characterisation of kink bands in vdW crystals. Kink bands are deformation features that are often visible in optical microscope images of exfoliated flakes of 2D materials appearing as straight line striations approximately 1 µm wide (Fig. 1a). These appear along ac or zz directions and are formed by basal plane dislocations^22,23^ due to applied thermal or mechanical stress. We have discovered that such defects predominantly consist of regions of perfect crystal lattice separated by boundaries where the basal planes are locally bent to the crystallographic twin angle (Eq. 1 and Table 1). However, it is clear that, when imaged at the atomic scale, these twin boundaries are not abrupt classical twins but delocalised over tens of atoms; in the boundary region the perfect stacking of the neighbouring crystals is disrupted (Fig. 1e, f). Atomic resolution on both sides of the boundary can also be used to confirm commensurate crystal stacking (Fig. 1e). However, this is often impossible to obtain in transmission electron microscope (TEM) or STEM images due to local strain effects. When this is not present a twin boundary must be identified from the quantisation of the slip vector (**L**_slip_), measured from the difference in the length of neighbouring basal planes across the boundary (Supplementary Note 2).

### Analysis of bend angles and crystal thickness

**Fig. 2:**
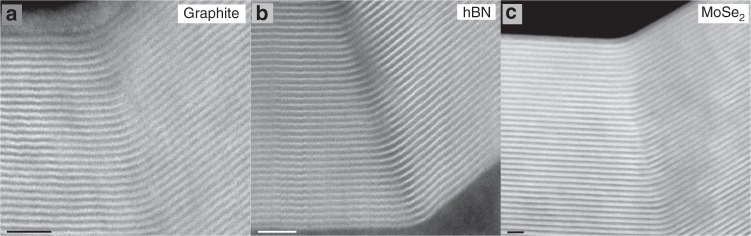
Cross-sectional HAADF STEM images of delocalised twin boundaries in 2D materials. **a** in graphite; **b** hBN; and **c** MoSe_2_. The viewing angle is down the armchair direction for all materials. This geometry is seen repeatedly in graphite kink bands as well as in vdW materials with different stacking preferences, interlayer adhesion and bending moduli. The bend angle in each material is dependent on its crystal lattice parameters (Table 1). All scale bars 2 nm

We have investigated the atomic morphology of bends, folds, and kink band ridges in graphene, hexagonal boron nitride (hBN) and molybdenum diselenide (MoSe_2_). Performing lattice resolution STEM imaging of mechanically deformed crystals allows measurement of bend angles and slip translation vectors for individual boundaries. This analysis has revealed that all three vdW materials contain abundant twin boundaries despite their different stacking preferences, interlayer adhesion and bending moduli (Fig. 2). The ac twin boundaries shown in Fig. 2 have measured bend angles, *θ*, of approximately 40°, 39°, and 30° for graphite, hBN and MoSe_2_, respectively, in good agreement with theoretical predictions for *θ*_*t*_ of 40.3°, 41.2° and 30.0° (Table 1). Importantly, all twin boundaries demonstrate a curved region of incommensurate stacking with widths of 1.3 nm, 1.1 nm, 1.4 nm for graphite, hBN and MoSe_2_, respectively. This delocalised interface is very different from conventional metallic twin boundaries, and perhaps explains why such features have not previously been identified as twin boundaries despite appearing in literature TEM images^21^.

A more statistical analysis of a large number of single grain boundaries has revealed that bend angles cluster within ± 18% of the ideal twin angle *θ*_*t*_ for all systems (for discussion see Supplementary Note 2). We propose that this variation in bend angle arises from the ability of the incommensurate region close to the twin boundary to accommodate significant local strain of the basal planes (for example, monolayer graphene is known to accommodate high elastic strains up to 1.5%^24^) and the relatively low stacking fault energy of these materials.

**Fig. 3:**
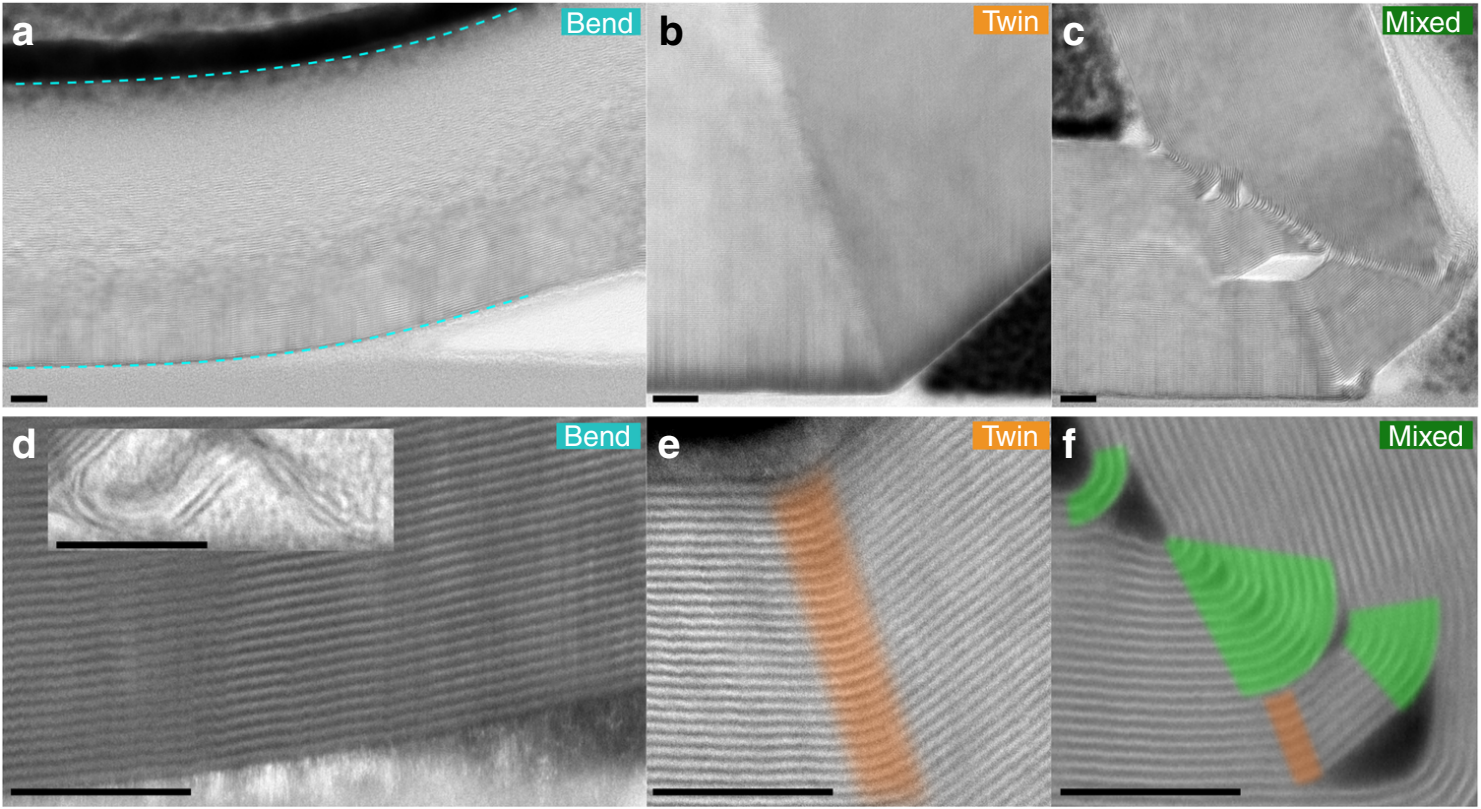
HAADF STEM images of different bending phenomena in graphite. **a**–**c** Bright field STEM images showing the microstructure of graphite for each bending mode: Bend, Twin and Mixed modes. The bend angle and number of basal planes in the crystal thickness for each are *θ* = 12°, *N*_1_ = 114 basal planes; *θ* = 40°, *N*_1_ = 559; and *θ* = 115°, *N*_1_ = 107, respectively. **d**–**f** HAADF STEM images demonstrating the different ways vdW crystals modify the arrangement of basal planes to accommodate strain induced by bending. The inset in **d** shows a 95° bend angle in a graphene bilayer. Areas of discrete twin boundaries are highlighted orange and areas of nanotube-like curvature are highlighted green. The mixed bending mode exhibits multiple discrete twins converging on a region of nanotube-like curvature and is only observed for the largest bend angles. All scale bars 5 nm

Figure 3 compares STEM images for sheets of graphite with different thicknesses subjected to increasing bend angle. At large bend angles we uncover an even more dramatic deviation from conventional twinning. Figure 3f reveals the first observation of a mixed bending mode where basal planes in the lower part of the crystal form separate discrete twin boundaries (highlighted orange) converging to an extended region of uniform curvature towards the bend apex (highlighted green). In the latter, the material consists of concentric incommensurate basal planes with uniform curvature, akin to multi-walled nanotubes (see Supplementary Note 2).

**Fig. 4:**
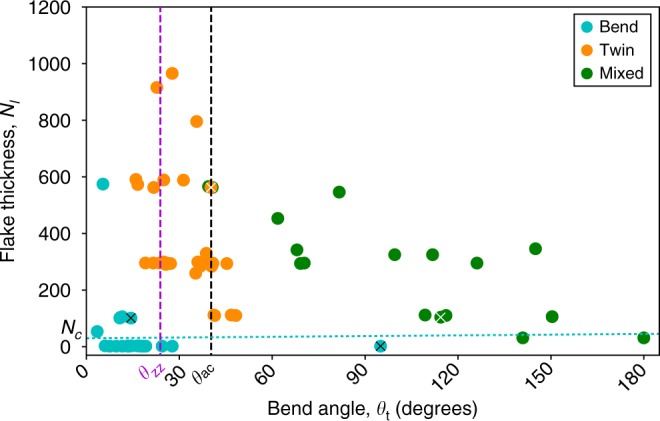
Plot of observed bending phenomena in graphite. The plot demonstrates the distribution of ‘Bend’, ‘Twin’ and ‘Mixed’ modes of deformation observed experimentally for different bend angles and thicknesses of crystal for both ac and zz bending. The dashed black and purple lines are the theoretically predicted ideal bend angles *θ*_*t*_ for ac and zz twins, respectively. The theoretical minimum thickness threshold for the twin or mixed modes is denoted by the dotted blue line. The data-points derived from the illustrative images in Fig. 3 are annotated with a cross. Bend mode (blue data points) are where *θ* < *θ*_*t*_ and for any thickness the crystal can accommodate the strain with gentle bending whilst remaining fully commensurate, and also where *θ* > *θ*_*t*_ but the flake is sufficiently thin that deformation results in nanotube-like bending of the basal planes. Twin mode (orange data points) is where above a critical flake thickness *N*_c_ and around or below *θ*_*t*_, discrete twin boundaries are found. The mixed mode (green data points) are where thicker flakes above *θ*_*t*_ display a third and more complex configuration combining twinning and bending

Looking at a large number of the bending phenomena experimentally as a function of the number of atomic layers *N*_1_ for the crystal flakes and their bend angle *θ* (Fig. 4) we can classify them as follows: (a) For all crystal thicknesses with small bend angles bending can be accommodated by strain in the lattice (indicated in blue and referred to as Bend data points, no twin boundary present in the microstructure). (b) At small crystal thickness (few layers), nanotube-like bending is observed to accommodate bending of any angle, even greater than 90° (also shown in blue and referred to as Bend data points, no twin boundary). (c) For all but the thinnest flakes, twin boundaries are observed for angles close to *θ*_*t*_, (indicated by orange data points and labelled Twin) and (d) a mixed bending mode is observed for larger bend angles (at least one twin as well as nanotube-like bending, labelled in green and referred to as Mixed data points).

Using numerical modelling combined with first-principles calculations for stacking fault energies and bending moduli (Supplementary Note 3), we can confirm our experimental observations. At small bend angles crystals of any thickness will remain commensurate by accommodating the shear strain through uniform nanotube-like bending of the basal planes, without using discrete local grain boundaries. For thicker flakes and bend angles approaching *θ*_*t*_, it is necessary to consider the balance between the energy required to bend the basal planes, and that associated with creating regions of incommensurate stacking and/or stacking faults. For nanotube-like bending the total energy scales as ~(*N*_1_)^2^+ln(*N*_1_), while for a discrete twin boundary the total energy scales as ~*N*_1_. As a result nanotube-like curvature is more energetically favourable for thin crystals (small *N*_1_) whereas discrete twinning becomes more favourable for thicker crystals (large *N*_1_). The thickness threshold, *N*_c_, at which nanotube-like curvature becomes unfavourable for graphite is predicted to be approximately 30 basal planes (blue dotted line in Fig. 4), in good agreement with experiment; the thinnest mixed-mode bending is observed in graphite flakes 31 basal planes thick.

For moderately thick flakes (*N*_1_ > *N*_c_) at angles significantly greater than the twin angle we find that it is energetically favourable for the crystal to form multiple twin boundaries and/or exhibit the mixed bending mode shown in Fig. 3c–f. For mixed mode deformation the nanotube-like structure (incommensurate basal planes with high curvature) is only favourable for a limited number of atomic layers (*N*_0_) closest to the bend apex. Further from the bend apex (plane index, *N* > *N*_0_) the defect splits into discrete lower angle twins that locally are similar to those described in Fig. 2. Our theoretical calculations predict an approximate transition thickness for graphite of *N*_0_ = 16 in reasonable agreement with the experimentally observed transition at *N*_0_ = 10 shown in Fig. 4 (Supplementary Note 3). The difference in these values likely arises from our calculations, which cannot account for in-plane strain^25^. The transition between the nanotube-like curvature and discrete twin boundaries is analogous to the appearance of radial corrugations in large-diameter multi-walled nanotubes^26^.

To the best of our knowledge, this is the first time such mixed mode bending has been observed and characterised. Small cracks are often seen at the interface between discrete twins and nanotube-like bending in all three materials. We speculate that the interface between nanotube-like bending and discrete twin regions may act as a weak point for nucleation of interlayer cracking; a process thought to be fundamental to exfoliation routes for 2D materials^5–8^. Crystals are also heavily damaged where twins along different crystallographic directions cross each other (such as at intersecting kink bands, Fig. 1b), which may lead to fragmentation of the crystals during exfoliation. Furthermore, we observed that larger delamination cracks are formed at angles greater than 3*θ*_*t*_ for crystals with thicknesses greater than *N*_c_ (Supplementary Notes 4 and 5). The absence of delamination cracks in thin crystals could help explain why liquid-phase exfoliation of 2D materials struggles to yield large area flakes thinner than ~7 layers ^5^.

### Anomalous twins to induce stacking faults

**Fig. 5:**
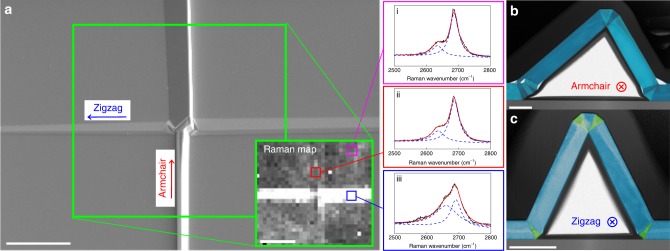
Twin boundaries creating unusual Raman-active stacking in graphite. **a** SEM image of two orthogonal kink bands intersecting in the crystal. Inset maps the Raman intensity ratio of the 2630 cm^−1^ and 2690 cm^−1^ peaks in the 2D peak. The ratio is drastically modified locally to the zz kink band (white contrast in inset). Scale bars 10 μm. Individual Raman 2D spectra are shown for (**i**) pristine graphite, (**ii**) the ac twin and (**iii**) the zz twin boundaries. **b** Large field of view bright field STEM image of the ac kink band. In this striation the basal stacking in each crystalline region is equivalent (Bernal stacked). False colour is used to highlight areas of lattice separated by a twin boundary. This ac kink band shows some delamination yet gives the same 2D Raman signal as pristine graphite. Scale bar 250 nm. **c** Large field of view bright field STEM image of the zz kink band. Regions of Bernal stacking are false coloured blue while small areas of crystal that do not exhibit Bernal stacking and are instead highly faulted or AA’ stacked are false coloured green. These areas are thought to contribute to the modification of the Raman 2D signal. Scale bar 250 nm

Finally, we demonstrate that zz twin boundaries in graphite can induce local stacking faults, and thus the Raman signature of zz kink bands can be used to determine crystallographic directions in graphite. Figure 5a shows a scanning electron microscope (SEM) image and Raman imaging of zz and ac direction kink bands meeting at 90° in a thick flake of graphite. Analysis of the shape of the Raman 2D band reveals significant changes associated with the zz kink band but not for the ac direction kink band. The disruption of the Raman peak can be explained if we consider the formation of a twin boundary through a series of lattice translations along the favoured crystal directions. A sequence of three zz ideal twin boundaries (each with the expected *α*/√3 slip translations along the perpendicular ac direction) theoretically produces a region of crystal with a complex stacking sequence AABBCC (derived in Supplementary Note 3). This is a highly unfavourable stacking configuration in graphite. We therefore propose that it is more energetically favourable to induce stacking faults in small regions of the crystal between pairs of twin boundaries^27^ (highlighted by the green shading on Fig. 5c). It is well established that a change in stacking in graphite has a strong effect upon the Raman signature, consistent with the changes we observe experimentally for the zz kink band (Fig. 5a and Supplementary Note 6)^22,28^. This frustrated stacking will not be present in hBN or the transition metal dichalcogenides where AA’ stacking is preferred^2^ but we note that 2D materials with similar stacking preferences to graphite may exhibit comparable lattice disruption.

## Discussion

In conclusion, we have presented an atomic scale analysis of stress induced twin and mixed mode boundaries in vdW crystals of 2D materials, revealing a hitherto unsuspected level of unusual behaviour that we have been able to analyse and understand as a whole for the first time. We have discovered that the deformation microstructure depends on the mechanical properties of the material, the crystal thickness and bend angle, and deformation features can be predicted from numerical and first principles calculations. Thin crystals present nanotube-like bending while thicker crystals show discrete twins at low angles or mixed mode deformation at high angles. We find that a weak interface exists in mixed-mode bending that may act as a nucleation site for exfoliation. Our results provide fundamental insights for understanding the mechanisms of exfoliation for 2D crystal flakes, which are crucial to the development and application of 2D materials. Moreover, the crystallographic bending features and local stacking variations observed here have important implications for the electronic properties of the materials. For instance, graphene has been predicted to form p-n junctions^29^ when bent and a similar effect in thicker films can partially account for observed modifications of the 2D Raman band^29,30^. It is also anticipated that transition metal dichalcogenides undergo significant change of their electronic spectrum on bending^31^, which could be exploited for sensors or flexible electronics applications.

## Methods

### Cross-sectional specimen preparation

Thin graphite, hBN and MoSe_2_ single crystal flakes were prepared on separate silicon substrates by mechanical exfoliation using adhesive tape^7^. For cross sectioning, the flakes were coated with 7 nm of amorphous carbon and 3 nm of gold-palladium alloy to protect them during imaging and milling. A FEI Helios Nanolab DualBeam 660 instrument (incorporating a SEM and a focused ion beam (FIB) in the same chamber) was used to image the flakes and produce cross sections. Large, uniform striations seen in almost all thick flakes were identified by SEM imaging and with tilting of the SEM stage.

Cross sections were prepared perpendicular to the striations within the flakes. A 2 µm thick platinum layer was deposited over the region of interest (ROI) using a gas injection system and by patterning with the Ga^+^ FIB. Large trenches are milled either side of the ROI to a depth of 5 µm using high current and high energy ions to leave a window comprising (ordered surface to substrate) platinum, Au/Pd, carbon, crystal striation, native silicon oxide and the silicon substrate. Once the FEI Easylift™ nanomanipulator was inserted and welded to the platinum layer with extra platinum, the window was cut free from the substrate and transferred to a pillar on a specialist OmniProbe™ TEM grid. The window was thinned to a TEM-transparent lamella with a thickness below 100 nm using 30 kV, 16 kV, 5 kV and 2 kV ion beam milling and polishing.

### Scanning tranmission electron microscope imaging

For high-resolution STEM imaging a probe side aberration corrected FEI Titan G2 80–200 kV was used with a probe convergence angle of 21 mrad, a HAADF inner angle of 48 mrad and a probe current of about 80 pA. To ensure the electron probe was parallel to the basal planes, the cross-sectional sample was aligned to the relevant Kikuchi bands of the silicon substrate and the 2D crystal.

The Raman spectra were obtained from the (0001) surfaces of the 2D crystals using a Renishaw inVia Raman system or Horiba LabRAM HR Evolution spectrometer, both equipped with HeNe lasers (*λ* = 633 nm), with the diameter of the laser spot on the crystal was estimated to be around 2 μm.

### Density functional theory

The density functional theory (DFT) calculations were performed using the periodic plane-wave basis set code VASP 5.35 and projector-augmented-wave potentials. The exchange–correlation functional is described by the revised Perdew–Burke–Ernzerhof (PBE) exchange model with the empirical dispersion correction of Grimme (DFT-D2). A plane-wave cut-off of 400 eV was used for the graphene and hBN layers and a value of 280 eV was used for the MoSe_2_ layers. We used a Monkhorst–Pack *k*-point grid of 11 × 11 × 1 per unit, which ensured that the bond lengths and energies converged to within 0.001 nm and 1 meV, respectively.

## Electronic supplementary material

Supplementary Information

Description of Additional Supplementary Files

Supplementary Movie 1

## Data Availability

All the original data are available from the corresponding author on request or can be obtained from the article’s listing on the University of Manchester’s PURE repository (www.pure.manchester.ac.uk).
