# Supplementary material for: Anomalous twin boundaries in two dimensional materials
Source: Nat Commun. 2018 Sep 5;9:3597. doi: 10.1038/s41467-018-06074-8 (PMC6125487; doi:10.1038/s41467-018-06074-8)
Supplement: Supplementary file 1 — Supplementary Information [file 41467_2018_6074_MOESM1_ESM.docx]

Anomalous twin boundaries in two dimensional materials

*Supplementary Information*

A. P. Rooney^1^, Z. Li^1,2^, W. Zhao^3,6^, A. Gholinia^1^, A. Kozikov^4,5^, G. Auton^2,4^, F. Ding^3,6^, R. V. Gorbachev^2,5^, R. J. Young^1,2,3^, S. J Haigh^1,2^*

1. School of Materials, University of Manchester, Manchester M13 9PL, UK
2. National Graphene Institute, University of Manchester , Manchester M13 9PL, UK
3. Institute of Textiles and Clothing, Hong Kong Polytechnic University, Hung Hom, Hong Kong
4. Manchester Centre for Mesoscience and Nanotechnology, University of Manchester, Manchester M13 9PL, UK
5. School of Physics and Astronomy, University of Manchester, Oxford Road, Manchester, M13 9PL, UK
6. Center for Multidimensional Carbon Materials, Institute for Basic Science (IBS-CMCM)/School of Material Science and Engineering, Ulsan National Institute of Science and Technology (UNIST), Ulsan 44919, Korea

Email: sarah.haigh@manchester.ac.uk

**Supplementary Figures**

**Supplementary Figure 1|The geometry and elements of deformation twinning. *K*_1_ is the twin or composition plane, *η*_1_ is the shear direction, *K*_2_ is the second undistorted plane and *η*_2_ is the direction lying in *K*_2_ that is rotated to *η’*_2_ but undistorted by the shear (Adapted with permission from** **John Wiley and Sons).**^1^

**Supplementary Figure 2|The structure proposed by Freise and Kelly**^2^ **of a twin in graphite as the result of a partial dislocation of Burgers’ vector a/3[10-10] or a/3[01-10] on each basal plane. (Reproduced with permission from The Royal Society).**


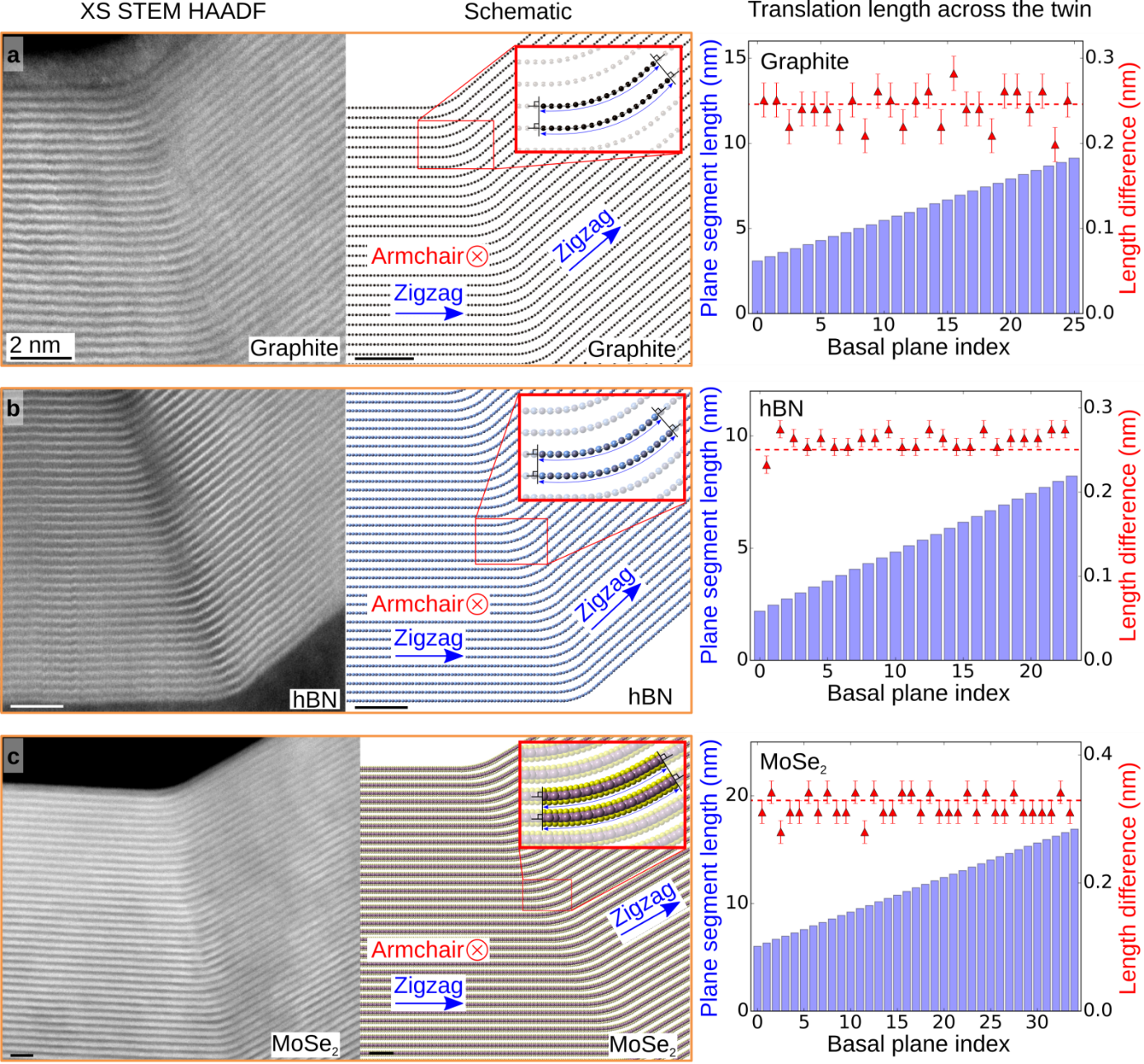


**Supplementary Figure 3|Cross sectional STEM images of ac direction twins in (a) graphite, (b) hBN, and (c) MoSe_2_**. **Left**, Lattice resolution HAADF STEM images of an ac twin boundary in each material. **Centre**, Atomic scale schematic showing the relative crystallographic orientations of the STEM image. Inset shows how the length of each plane is measured across the curved region of the twin boundary. **Right**, (blue bar chart) The measured path length of the curved boundary region for each plane shown in the HAADF STEM image. Taking the difference in path length between neighbouring basal planes gives the translation length across the boundary that is found to be consistent for the whole thickness of the crystal (length difference plotted as red triangles). The expected value of path difference, consistent with one zigzag translation, is denoted by the dotted red line. All scale bars 2 nm.

**
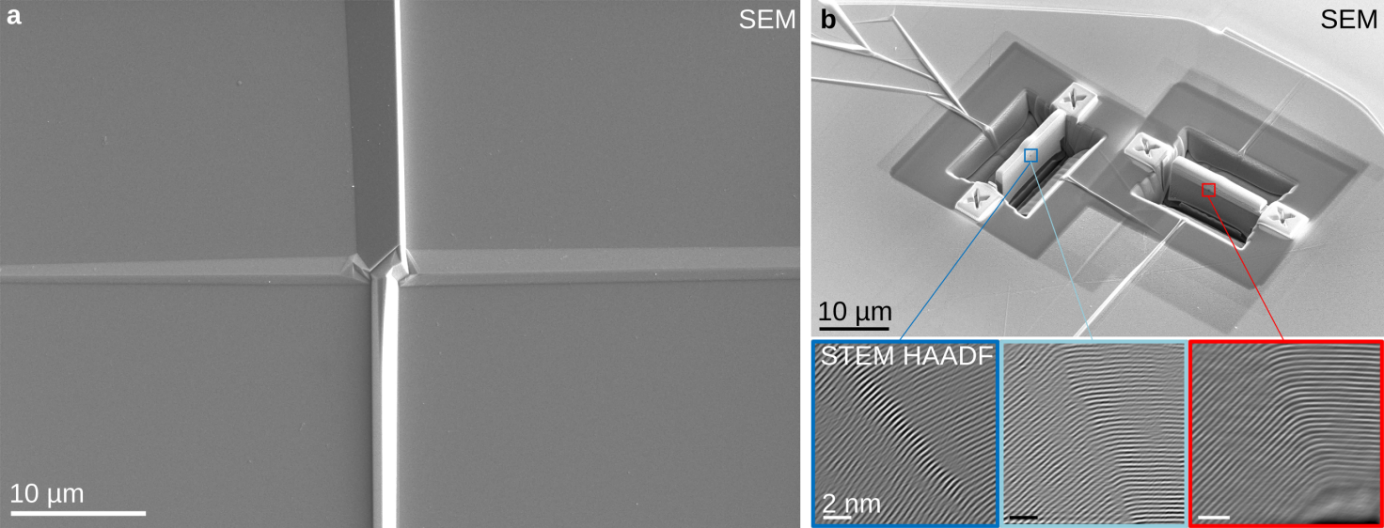
**

**Supplementary Figure 4|SEM and HAADF STEM images of orthogonal twin striations in graphite. a**, SEM plan view image of two orthogonal twin striations in graphite meeting. **b**, Top: SEM oblique view of two similar graphitic twin striations. Lamellae cutting perpendicular to the striations have been milled by FIB and are awaiting lift-out and transfer onto a TEM grid. Bottom: A three filtered HAADF STEM images from each lamella. By measuring the twin angle and the slip translation length of the twin boundary for each we can determine the crystallographic direction of the twinning striation.

**
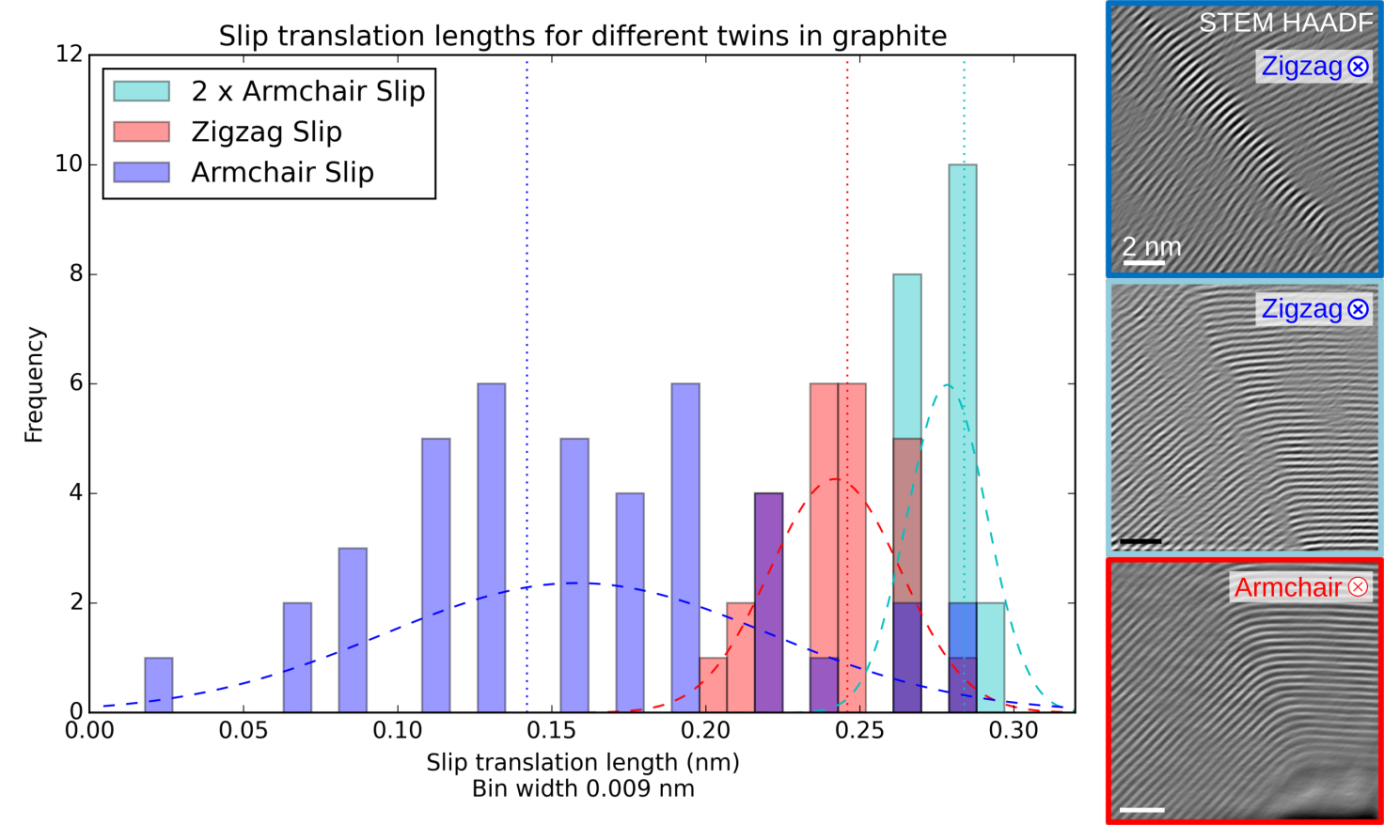
Supplementary Figure 5|Determining the crystallographic direction of the twinning striations in Supplementary Figure 3. Left:** Histogram showing the slip translation length for basal planes in each of the three images shown **Right**. The colours bordering each image correspond to the colours of the three datasets in the histogram. The dotted vertical lines are, from left to right, the lengths of one ac, one zz and two ac translations in graphite taken from Table 1 (see main text). Each distribution can be fitted by a Gaussian (dashed bell curves) to get the mean and standard deviation of each dataset. The mean values are all within 0.02 nm of the ideal translation lengths and, coupled with twin angle measurements, we can determine the direction of the slip and therefore the twin direction for each image (labelled top right in each HAADF image). The standard deviation of the distributions decreases for increasing slip translation length. All scale bars 2 nm.


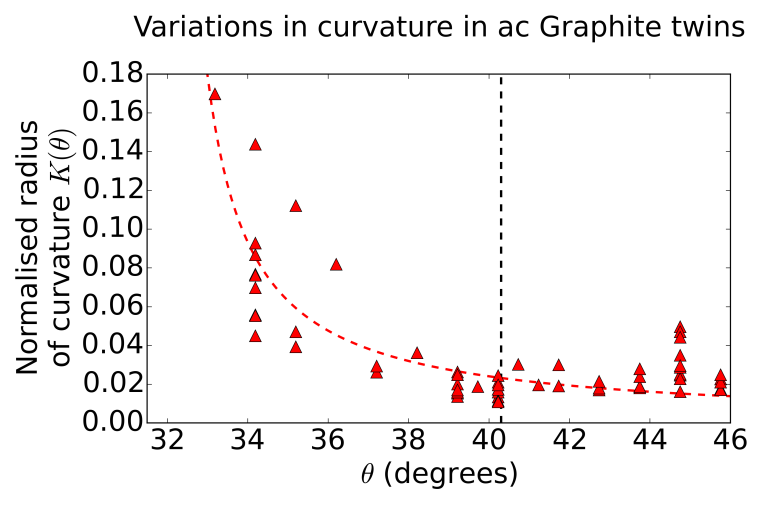


**Supplementary Figure 6|** The relationship between the basal plane radius of curvature K(θ) and twin angle for ac twins in graphite. The ideal twin angle is shown as the dashed black line at 40.3°. The distribution is measured from different HAADF STEM images and is fitted with a simple asymptote to estimate the onset angle for the ac twin in graphite.

**

**

**Supplementary Figure 7| Inducing bending phenomena in graphite using a micromanipulator in a SEM.** The micromanipulator was moved across the surface of a graphite flake, cleaving the top-most layers. This resulted in the different bending structures in graphite seen around the tip of the micromanipulator.


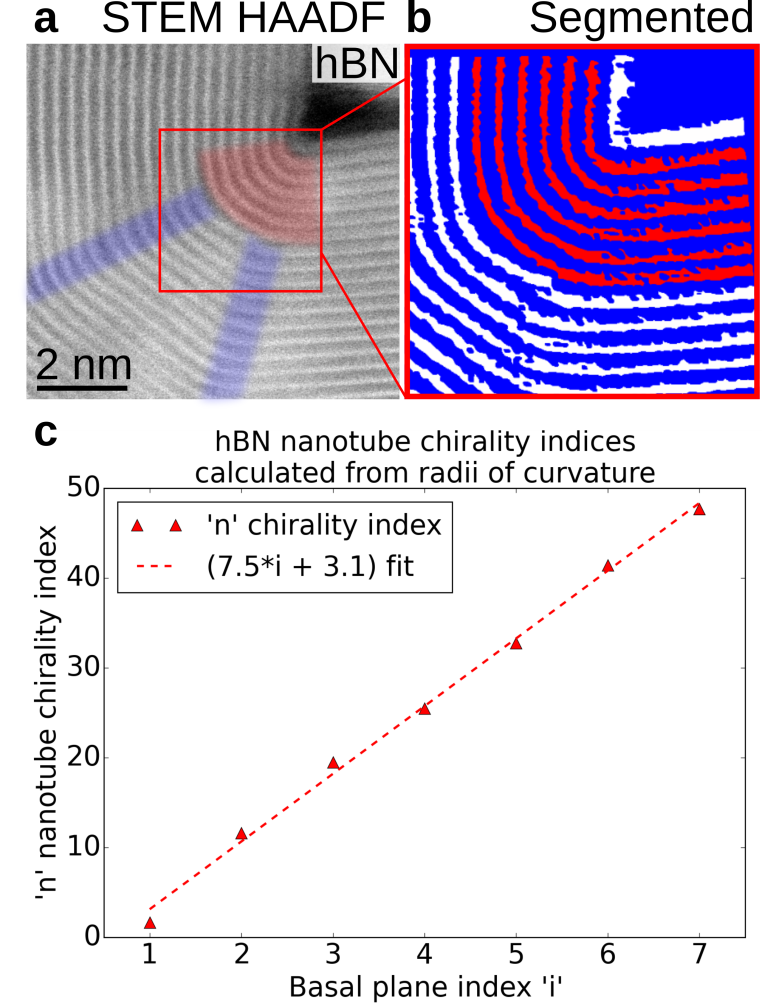


**Supplementary Figure 8| Determining the equivalent nanotube chirality for basal planes in hBN.** **a**, HAADF STEM image of a high shear bend in hBN. The discrete twins are highlighted blue and the region of nanotube-like curvature is shown red. **b**, smaller field of view image of the region highlighted in **a**. The image is composed of segmented basal planes, some highlighted red to show nanotube-like curvature. Each red plane is assigned an equivalent nanotube chirality, from top to bottom: (11, 0); (19, 0); (27, 0); (35, 0); (43, 0); and (51, 0). **c**, plot of calculated nanotube chirality for each basal plane. A line of best fit allows us to estimate the chirality of the basal planes and the lower limit of curvature for this type of bending.

**Supplementary Figure 9|Schematic illustration of nanotube-like (NT) bending.** The separation of the basal planes is *d* and the same as in the undeformed crystals but the interaction between the planes is weakened because of the loss of registry between the planes due to the presence of stacking faults. The radius of curvature of the planes increases with increasing layer number, *l*.

**Supplementary Figure 10 |Schematic illustration of Mode 2 discrete twinning.** This process leads to a larger layer-layer distance within the bend and hence a greater reduction of interlayer interaction energy than for NT bending.


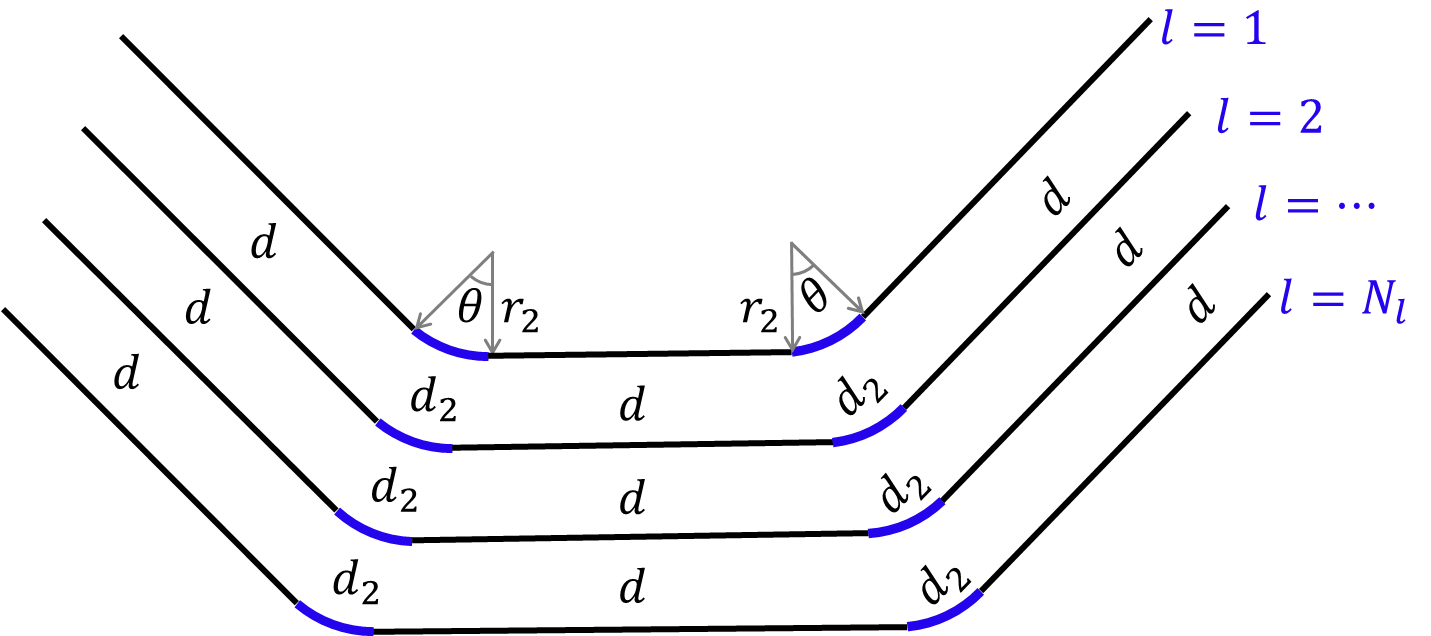


**Supplementary Figure 11|Schematic diagram of a double discrete twin.** This is formed by the breakdown of a discrete twin with an angle of $2\theta$ into two twins each with a twin angle of $\theta$.

**Supplementary Figure 12|DFT analysis of the energetics for the separation (upper graph) and sliding (lower graphs) of layers within the 2D materials** graphite, boron nitride and molybdenum diselenide. Calculations performed by considering bilayer material. The parameter $E_{i}$ is the overall van der Waals interaction energy between the two layers.

**(a) NT bending: when $\boldsymbol{N}_{\boldsymbol{l}}\boldsymbol{=10}$,** Å.

**(b) DT bending: when $\boldsymbol{N}_{\boldsymbol{l}}\boldsymbol{=34}$,**

**Supplementary Figure 13|Variation of the energy per unit width of the ac twins in graphite as a function of radius of curvature,** ($r_{1}$ for NT bending and $r_{2}$ for DT bending). (a) NT bending (*N_l_* = 10), gives minimum total energy (plotted black) at $r_{1}=1.29 \mathrm{nm}$ and (b) DT (*N_l_* = 34) gives minimum total value at $r_{2}=1.69$ nm.

**Supplementary Figure 14|Comparison of the measured and DFT predicted values for the minimum radii of curvature for ac twinning,** (a) Nanotube-like (NT) bending and (b) Discrete twinning (DT). Experimental values measured directly from STEM images.

**NT Bending:**

$\boldsymbol{E}_{\boldsymbol{vdW}\mathbf{1}}\boldsymbol{\sim}\left( \boldsymbol{N}_{\boldsymbol{l}} \right)^{\mathbf{2}}$

$\boldsymbol{E}_{\boldsymbol{bend}\mathbf{1}}\boldsymbol{\sim}\ln\left( \boldsymbol{N}_{\boldsymbol{l}} \right)$

$\boldsymbol{E}_{\boldsymbol{tot}\boldsymbol{1}}\boldsymbol{\sim}\left( \boldsymbol{N}_{\boldsymbol{l}} \right)^{\mathbf{2}}\mathbf{+}\ln\left( \boldsymbol{N}_{\boldsymbol{l}} \right)$

**DT bending:**

$\boldsymbol{E}_{\boldsymbol{vdW}\mathbf{2}}\boldsymbol{\sim}\boldsymbol{N}_{\boldsymbol{l}}$

$\boldsymbol{E}_{\boldsymbol{bend}\mathbf{2}}\boldsymbol{\sim}\boldsymbol{N}_{\boldsymbol{l}}$

$$\boldsymbol{E}_{\boldsymbol{tot}\boldsymbol{2}}\boldsymbol{\sim}\boldsymbol{N}_{\boldsymbol{l}}$$

**Supplementary Figure 15|Analysis of the change in energy per unit width as a function of the number of layers, *N*_l_ for the different twinning modes in graphite** **with traces in the ac direction.**

**Supplementary Figure 16| Variation of *α*_2_ with *ϕ* calculated using DFT.** The examples given are for discrete twinning with traces in the ac direction for graphite, hBN and MoSe_2_.


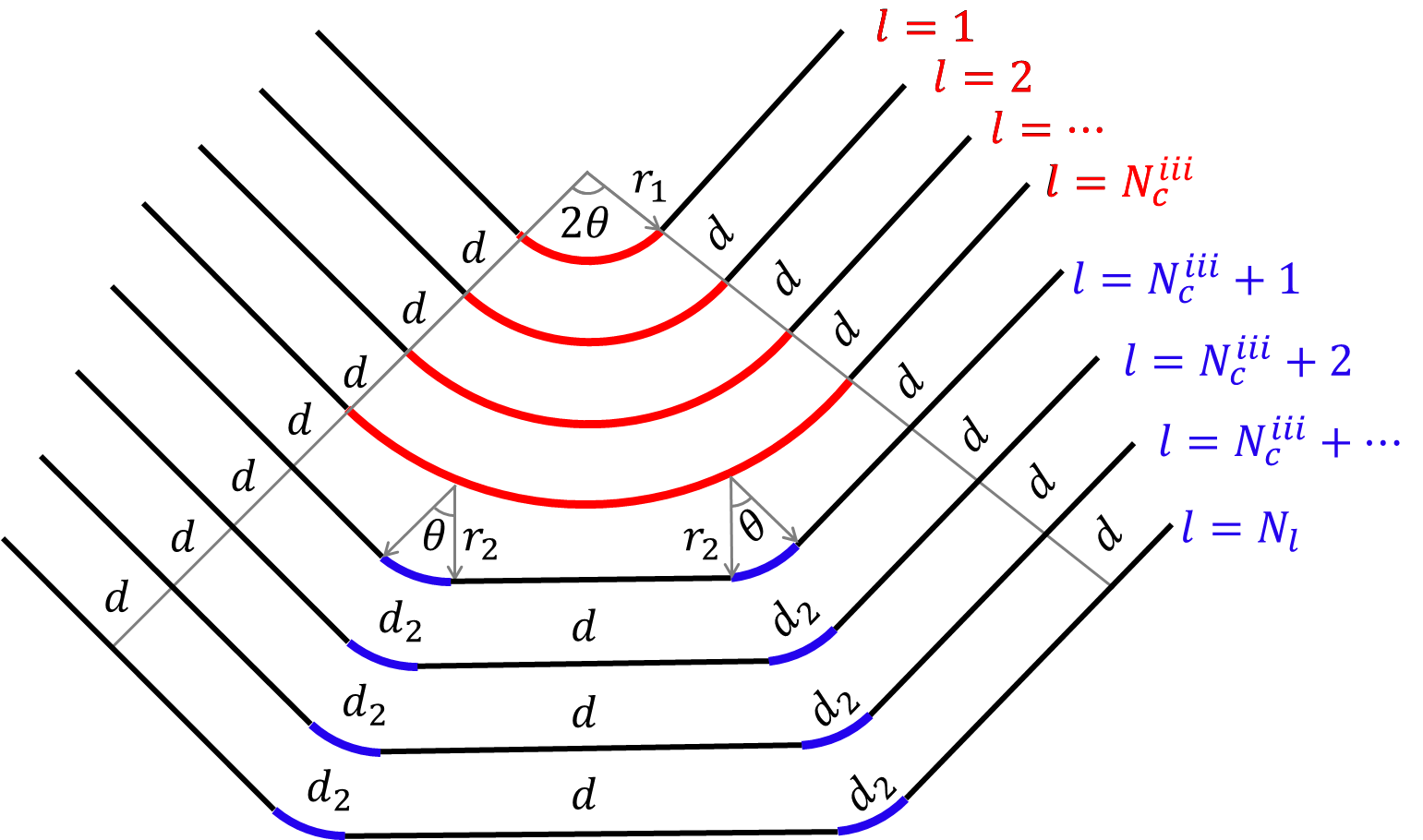


**Supplementary Figure 17|Examples of hybrid twin boundaries made up of a mixed mode of NT bending and DT twinning.**

**Supplementary Figure 18|Analysis of the change in energy per unit width as a function of the number of layers, *N*_l_, for the twinning modes for twinning with an ac direction trace in graphite.** It can be seen that the energy for a single discrete twin with *θ* = 84° is significantly higher than that of two twins with *θ* = 42° and so the high angle twin will always split into two lower angle ones. It can also be seen that for this double twin, ${N_{c}=30 \mathrm{and} N}_{0}=16.$


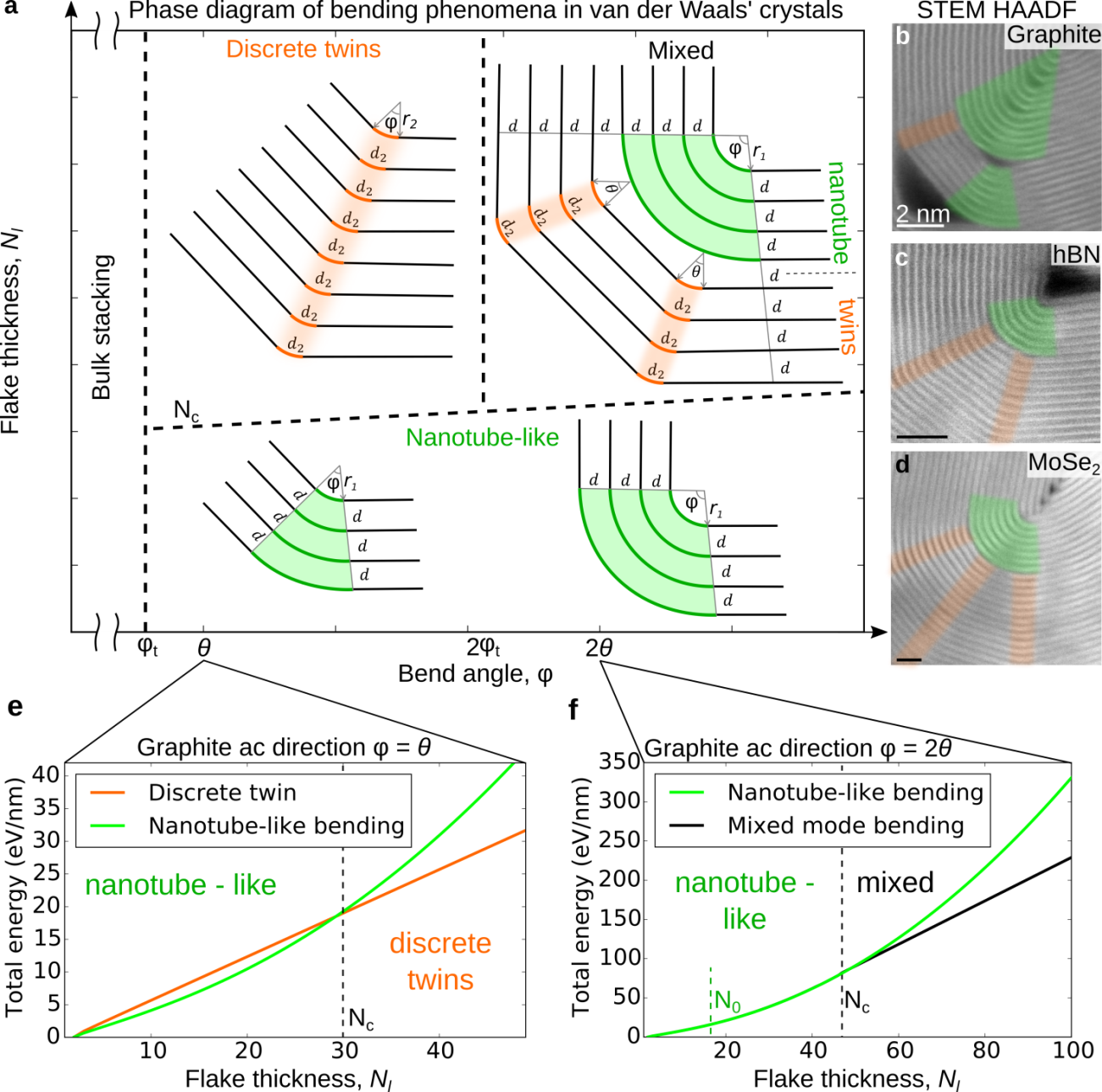


**Supplementary Figure 19|** **Summary** **phase diagram of bending phenomena in van der Waals’ crystals. a,** Phase diagram containing schematics demonstrating the different ways van der Waals’ crystals modify the arrangement of basal planes to accommodate the strain induced by a bend angle ϕ. For flakes of any thickness, if ϕ < ϕ_t_ the crystal can accommodate the strain with gentle bending whilst being fully commensurate. For thin flakes, any ϕ > ϕ_t_ results in nanotube-like bending of the basal planes. Above a flake thickness *N_c_* and a bend angle ϕ_t_ we find that two configurations are possible, depending on the value of ϕ. If ϕ_t_ < ϕ < 2ϕ_t_ the boundary will consist only of discrete twins (see Figure 2). The competing energetics of nanotube-like curvature (green line) and discrete twins (orange line) are plotted in **e** and allow us to predict the thickness threshold *N_c_* for graphite ac kinks. If ϕ is larger than 2ϕ_t_ we observe a third configuration, which we have named the ‘mixed bending mode’, for thick flakes. Mixed bending mode exhibits multiple discrete twins converging on a region of nanotube-like curvature and is only observed in highly strained thick flakes. **f** plots the competing energetics of nanotube-like curvature (green line) and mixed mode bending (black line) for graphite ac kinks. **b, c, d,** Lattice resolution HAADF STEM images of mixed mode bending observed in a range of van der Waals’ crystals. Areas of discrete twins are highlighted orange and areas of nanotube-like curvature are highlighted green. All scale bars 2 nm.

**Supplementary Figure 20|Summary of the crystallographic stacking in crystals of 2D materials.**

Consider sliding in the armchair direction:

1. If the second B layer slides by *a*/√3, B sites become C sites. The third A layer then slides by 2*a*/√3 and A sites become C sites. The fourth layer then slides by 3*a*/√3 and B sites become B sites. The overall stacking becomes ..ACCBBA.., i.e. a stacking fault on every other layer.
2. If the second B layer slides by 2*a*/√3, B sites become A sites. The third A layer then slides by 4*a*/√3 and A sites become B sites. The fourth layer then slides by 6*a*/√3 and B sites become B sites. The overall stacking becomes ..AABBCC.., i.e. a stacking fault on every other layer.
3. Bernal stacking ..ACAC.. can be retained by sliding by *a*/√3 and 2*a*/√3 on alternate layers.

**Supplementary Figure 21|Summary of stacking issues for armchair direction sliding in AB Bernal stacked graphite.**


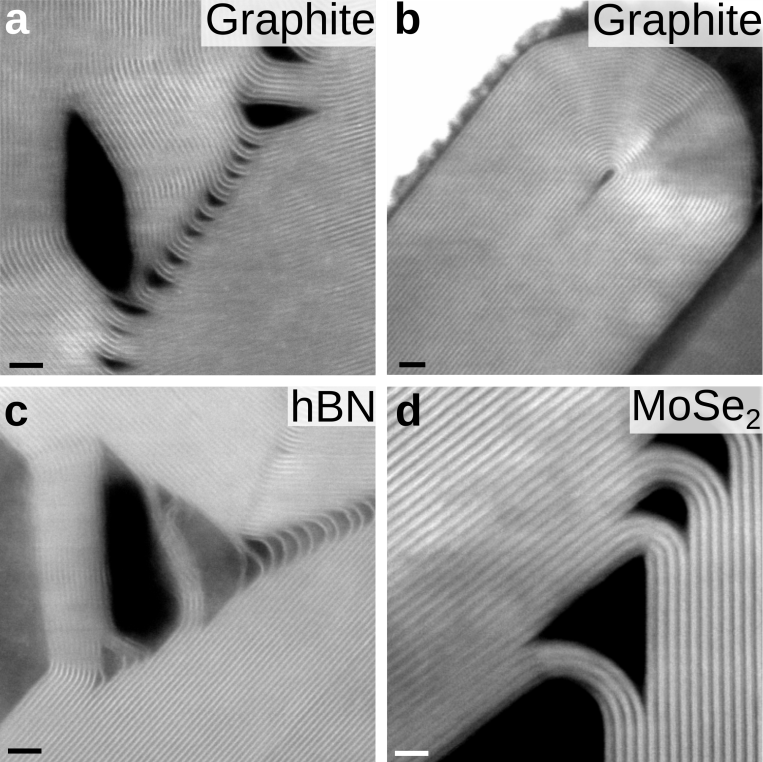


**Supplementary Figure 22|HAADF STEM images showing crack growth and other phenomena in highly strained crystals. a, c, d,** In highly strained regions, groups of basal planes perform multiple translations to form large cracks in ac graphite, hBN and MoSe_2_. The thickness of each group of basal planes ranges from 2-4 monolayers and have nanotube-like curvature. **b**, ac graphite with a bend angle of 180°. The flake has folded back upon itself and stacks perfectly outside the twin region. Within the bend there are five discrete twins which converge to homogenous nanotube-like curvature. All scale bars 2 nm.

**Supplementary Figure 23. The role of twinning in exfoliation**. **a** Optical micrograph of a thin crystal of graphite subjected to deformation and exfoliation showing twins and cracks (red arrows) with traces parallel to the zig-zag and armchair directions. STEM micrograph of a transverse section through a highly deformed region of a thin MoSe_2_ single crystal showing twins and exfoliated layers at **b** lower and **c** higher magnification. The SEM images of a junction of twins at **d** low and **e** high magnification. **f** SEM image of a junction of twins that cause the whole flake at the top surface to delaminate.

**Supplementary Figure 24***.* **Optical micrograph of crossing twins along with Raman spectra taken in scans across two orthogonal twins.**

**Supplementary Figure 25. Raman spectra of pristine graphite, ac twin and zz twin. Insets are M and G* band, respectively.**

**Supplementary Tables**

**Supplementary Table 1|Twinning parameters calculated from DFT analysis for ac and zz direction twins in graphene, hBN and MoSe_2_.**

| **Material** | **Graphene** | | | | **hBN** | | **MoSe_2_** | |
| --- | --- | --- | --- | --- | --- | --- | --- | --- |
| **Direction of twin trace** | **ac** | **zz** | | | **ac** | **zz** | **ac** | **zz** |
| *β* (eV) | 0.758 | 0.757 | | | 0.491 | 0.473 | 4.543 | 4.645 |
| *L*_slip_ (nm)* | 0.2464 | *a*/√3 | 2*a*/√3 | 3*a*/√3 | 0.2504 | 0.432 | 0.329 | 0.555 |
| *e*_0_ (meV/unit cell) | 50.80 | 50.80 | | | 66.833 | 66.833 | 81.315 | 81.315 |
| *e*_0_ (eV/nm^2^) | 1.9360 | 1.9360 | | | 2.4616 | 2.4616 | 2.6720 | 2.6720 |
| *c* (nm)* | 0.6711 | 0.6711 | | | 0.6661 | 0.6661 | 1.229 | 1.229 |
| *c* (nm) - DFT | 0.6460 | 0.6460 | | | 0.6194 | 0.6194 | 1.2652 | 1.2652 |
| *e*_0_-*e*_1_ (meV/atom) | 3.419 | 1.442 | 6.792 | 4.768 | 7.990 | 11.478 | 25.390 | 29.827 |
| *α*_1_ | 0.933 | 0.972 | 0.866 | 0.906 | 0.880 | 0.828 | 0.688 | 0.633 |
| *θ* (°)* | 40.3 | 23.9 | 46.0 | 64.9 | 41.2 | 65.9 | 30.0 | 48.6 |
| *θ* (°) - DFT | 43.6 | 25.2 | 50.4 | 75.6 | 46.9 | 81.2 | 30.1 | 52.1 |
| *d*_2_(θ) (nm) | 3.479 | 3.310 | 3.569 | 4.087 | 3.375 | 4.079 | 6.550 | 7.041 |
| *e*_0_-*e*_2_ (meV/atom) | 4.025 | 0.629 | 6.206 | 21.005 | 6.784 | 33.481 | 7.380 | 28.792 |
| α_2_(θ) - (DFT) | 0.921 | 0.988 | 0.878 | 0.587 | 0.898 | 0.499 | 0.909 | 0.646 |
| *α*_1_*α*_2_ - (DFT) | 0.859 | 0.960 | 0.760 | 0.531 | 0.791 | 0.413 | 0.625 | 0.409 |

* Parameters determined from experimental crystallographic data.

**Supplementary Table 2|energetics of the different twinning modes**

| **Mode** | **NT** | **DT** | **Mixed mode bending** |
| --- | --- | --- | --- |
| **vdW interaction** | $\sim\left( N_{l} \right)^{2}$ | $\sim N_{l}$ | $\sim C_{1}\left( N_{c}^{iii} \right)^{2}+C_{2}(N_{l}-N_{c}^{iii})$ |
| **Bending energy** | $\sim\ln\left( N_{l} \right)$ | $\sim N_{l}$ | $\sim C_{3}\ln\left( N_{c}^{iii} \right)+C_{4}(N_{l}-N_{c}^{iii})$ |
| **Total** | $\sim\left( N_{l} \right)^{2}$+$\ln\left( N_{l} \right)$ | $\sim N_{l}$ | $\sim C_{1}\left( N_{c}^{iii} \right)^{2}+C_{3}\ln\left( N_{c}^{iii} \right)+C_{5}\left( N_{l}-N_{c}^{iii} \right)$ |

**Supplementary Note 1. Crystal Twinning**

Twinning in crystals has been reviewed extensively by Kelly and Knowles^1^ however for clarity we will briefly summarise the geometry of twinning here and how this relates to the work presented in the main manuscript. Twinning is a deformation mechanism whereby part of a crystal is sheared relative to the parent crystal in a unique shear direction, forming a twin boundary across which there is a mirror image orientation relationship between the twinned and untwinned (parent) region. The geometry of the simple shear associated with twinning, along with the definition of the twinning elements, is shown in Supplementary Figure 1. The twin plane *K*_1_ is the plane that is neither distorted nor rotated by the shear in the shear direction **η_1_**. The plane that contains both *η*_1_ and the normal to *K*_1_ is known as the plane of shear, *S*. It can be seen that the direction **η_2_** that lies in the plane of shear is rotated but undistorted by the twinning process. Likewise the second undistorted plane, *K*_2_, is rotated but undistorted by the twinning process. The magnitude of the shear, *s*, is therefore defined as $s=2\tan(\theta/2)$ where *θ* /2 is the angle between **η_2_** and the normal to *K*_1_.

Twinning deforms the crystal such that the Bravais lattice is reproduced in a specific new orientation and for high-symmetry crystals (e.g. cubic and hexagonal) the four elements *K*_1_, *K*_2_, **η_1_** and **η_1_** are generally all rational. Twins are conventionally classified in terms of the two elements, *K*_1_ and **η_1_**.

The twins that form most readily in crystals are generally those that involve the lowest shear, *s*, but a number of other considerations also have to be taken into account. For example, it is not sufficient that a twin merely reproduces the Bravais lattice and atom movements (shuffles) may be needed to reproduce the crystal structure^1^. Additionally in crystals containing covalently-bonded molecules, such as polymers, twins involving a low shear strain predicted from knowledge of the crystal structure do not form if they would result in the breaking of molecules^3,4^.

**Supplementary Note 1.1: Twinning in Graphite**

Extensive research upon the properties of graphite was undertaken in the 1950s and 1960s, as a consequence of its use as a moderator in nuclear power generation. Seminal work upon deformation twinning in graphite single crystals was undertaken by Freise and Kelly^2^ who showed that deformation twinning could take place: a process that involved bending of the (0001) basal plane about the $\langle1\bar{1}00\rangle$zigzag direction by an angle of the order of 20°. The habit plane of the twins was $\{11\bar{2}1\}$and, although they had no direct experimental evidence, Freise and Kelly^2^ calculated that the shear direction was most likely in the $\langle11\bar{2}0\rangle$armchair direction. Their optical micrographs of the $\text{(0001)}$ surfaces of deformed crystals show lines at 60°/120° degrees to each other, parallel to the three $\langle1\bar{1}00\rangle$directions. Kelly and Knowles^1^ reported the elements of the twin to be:

*K*_1_ = $\{11\bar{2}1\}$, *η*_1_ = , *K*_2_ = $(\text{0001)}$, *η*_2_ = $\langle11\bar{2}0\rangle$

with a magnitude of shear, *s*, of 0.367. This type of twin is classified as a $\{11\bar{2}1\}$ twin although it can be better envisaged as a rotation of the $(\text{0001)}$ basal planes by an angle of *θ* = 21° involving a shear of the basal planes in the $\langle11\bar{2}0\rangle$ armchair direction within the twin. The trace of the twin on the (0001) basal plane of a graphite crystal is parallel to the $\langle1\bar{1}00\rangle$zigzag (zz) direction. Hence we will term it a zz twin.

Freise and Kelly^2^ suggested that such a twin could accommodate atomic shuffles required to reproduce the Bravais lattice by a shear displacement of alternating (0001) basal planes by $\frac{1}{3}a[10\bar{1}0]$ and $\frac{1}{3}a[01\bar{1}0]$ as shown in Supplementary Figure 2. It was known that partial dislocations with Burgers vectors of this type exist in the basal planes of graphite. Since walls of such partial dislocations constitute a tilt boundary it was envisaged that the observed lateral growth of the twin could take place by the glide of a wall of partial dislocations into the matrix. It should be noted that this proposed structure leads to a change from AB stacking on one side of the twin to AC stacking on the other side of the boundary^2^.

A later study by Thomas and coworkers^5^ showed that unusual twins could also be found, particularly in thin graphite crystals, that intersected the common ones found by Freise and Kelly^2^ by angles of 30° and 90°. They presumed that these unusual twins had *K*_1_ planes of $\{1\bar{1}01\}$. The four elements of this twin must therefore be as follows:

*K*_1_ = $\{1\bar{1}01\}$, **η_1_** = $\langle11\bar{2}0\rangle$, *K*_2_ = $(\text{0001)}$, **η_2_** = $\langle1\bar{1}00\rangle$

and the shear, s = 0.734. This type of twin would be classified as a $\{1\bar{1}01\}$ $\langle11\bar{2}0\rangle$ twin although it can be better envisaged as a rotation of the $(\text{0001)}$ basal planes by an angle of 2*θ* = 40.3° involving a shear of the basal planes in the $\langle1\bar{1}00\rangle$ zigzag direction within the twin. The existence of such twins had been postulated earlier by Kennedy^6^. The trace of the twin on the (0001) basal plane of a graphite crystal will be parallel to the $\langle11\bar{2}0\rangle$ armchair (ac) direction. Hence we will term it an ac twin.

**Supplementary Note 2. Determining the Crystallographic Nature of Graphite Twins**

The crystallographic viewing direction of a crystalline specimen is traditionally derived from a transmission electron microscope (TEM) diffraction pattern or the Fourier transform of a lattice resolution TEM image, both of which map real space lattice information in the frequency domain. These methods require a large amount of high quality crystal to be present in the region of interest (ROI) to get a favourable signal to noise ratio and allow quantitative analysis. In the field of thin 2D materials these requirements are not always realised when looking at buried interfaces using cross sectional imaging.^7,8^

Here we have developed an approach where, using advanced image filtering and segmentation techniques, we are able to derive the crystallographic nature of twin boundaries in van der Waals crystals. Image processing was used in order to remove image noise such that continuous sections of basal plane could be successful segmented (recognised as separate lines by the software algorithm) and all useful information is retained. Gatan’s Digital Micrograph software was used to apply bandpass (1.5 – 7 nm^-1^) and radial filters to each image’s fast Fourier transform (FFT) to remove high frequency noise and information not associated with the curved basal planes. After Fourier filtering further, processing was performed using Hyperspy, NumPy, Matplotlib and Scikit-image python libraries.^9–12^ Adaptive thresholding was used to create a binary image, then particle detection and size thresholding allowed each basal plane to be identified as a separate object.

The length of each basal plane was then measured by applying masks to the two crystals, well away from the twin boundary and perpendicular to the basal planes in order to constrain the region of interest. Medial axis skeletonization was used to isolate the centre of the basal plane: the width of each basal plane was eroded until only a single chain of pixels comprising the centre of the plane remained. Gaussian filtering (10 pixel width) can help this process by smoothing the edges of a basal plane to eliminate branching. The number of pixels in a ‘skeleton line’ were counted to find the length of the basal plane in pixels. The images were then independently calibrated using the bulk lattice basal plane spacing from areas of the specimen with normal stacking free from defects. This calibration was then used to convert the number of pixels in the skeletonized line to the length of the basal plane in nanometres, as detailed in Supplementary Figure 3.

The radius of curvature of each basal plane was found by isolating the medial axis skeletonized points on the curve using masking. The points were fitted with a circle and the radius of curvature determined from the fit output. The length of the curved section of the basal planes was also determined by applying a Hough transform to determine the angle between straight sections of the same basal plane. For ac direction twins the mean length of the curved section was: 1.3 nm ± 0.2 nm for graphite, 1.1 nm ± 0.2 nm for hexagonal boron nitride (hBN), and 1.4 nm ± 0.3 nm for molybdenum disulphide (MoSe_2_).

**Supplementary Note 2.1: Distinguishing Armchair and Zigzag Twins in Graphite**

By taking the difference in length of neighbouring skeletonized basal planes, the slip translation length can be determined for the twin boundary (Supplementary Figure 3). Two orthogonal twinning striations were identified in a thick graphite flake on a silicon substrate after mechanical exfoliation. Distinguishing armchair from zigzag directions is not possible from optical or SEM images and consequently the twinning directions for the individual twins were unknown. However different twinning directions will have characteristic slip translation lengths (see Supplementary Note 1) and here we demonstrate that this can be used to determine the orientation of the twins from cross sectional HAADF STEM images. Knowing the twin directions it was possible to determine which of the twinning directions enhanced the Raman 2D peak in graphite (see Figure 4 in main text).

Lamellae were extracted from the twin striations using the FIB *in situ* lift-out method^7,13^ and imaged with HAADF STEM. A selection of images from each twin striation are shown bottom right in Supplementary Figure 4. Image processing to determine the slip translation length was carried out on each image and the slip translation length for each basal plane was found to correspond to a distribution about a crystallographic vector (i.e. multiples of the zz or ac translations, see Table 1 main text) (Supplementary Figure 5). Each distribution was fitted with a Gaussian curve to determine the mean and standard deviation slip translation length. These were found to be 0.16 nm ± 0.06 nm, 0.24 nm ± 0.02 nm and 0.28 nm ± 0.01 nm for the blue, red and cyan bordered HAADF STEM images, respectively. The mean values allow each image to be assigned a slip translation vector, all being within 0.02 nm of their corresponding ideal value calculated theoretically (Table 1 main text). These slip vectors were ac (0.14 nm), zz (0.25 nm) and two ac translation vectors (0.29 nm) for the blue, red and cyan bordered HAADF STEM images, respectively. The twin direction for each image is orthogonal to the slip direction (labelled on each image in Supplementary Figure 5).

Supplementary Figure 5 shows that the standard deviation of the slip translation decreases with increasing slip translation length. This trend can be explained in terms of the shear forces acting upon the basal planes about the boundary. Larger slip translations correspond to smaller angles and thus the compressive forces on the basal planes within the twin will be greater. We postulate that a compression of the crystal acting to decrease the interlayer separation makes any deviation from atomic registry increasingly unfavourable energetically, and consequently increases the tendency for perfect slip translations which maintain ideal stacking of the basal planes. For low shear twin boundaries (e.g. the ac slip (zz twin direction) boundary shown royal blue in Supplementary Figure 5) the forces are relatively weak and the slip can take a range of values since deviations from perfect stacking only produce a slight increase in energy. For higher shears with smaller twin angles, the energy minimisation of atomic registry becomes increasingly important, resulting in tighter distributions for the zz slip and 2 x ac slip twins, shown red and cyan.

**Supplementary Note 2.2: Angular Tolerance of ac Graphite Single Twins**

Pronounced deviation from perfect twinning is not only reserved for low shear twins. We find the radius of curvature changes markedly on deviating from the ideal twin angle in ac graphite. This ideal twin angle can be calculated from the slip translation length, **L­_slip_**, as:

$\theta=2 {tan}^{-1}\left[ \frac{\mathbf{L}_{\mathbf{slip}}}{c} \right]$ (Supplementary Equation 1)

For a graphite ac twin, where the slip translation length is one zz translation = 0.25 nm, the ideal twin angle is 40.3°. Many STEM HAADF images of ac twin boundaries in graphite were analysed to determine the relationship between the basal plane radius of curvature and the twin angle. This data is plotted in Supplementary Figure 6, where each red triangle corresponds to an individual basal plane. The ideal twin angle, 40.3°, is denoted by a black dashed line. The images held basal planes with twin angles over a range of ~40.3 ±7°. Twin angles larger than the ideal twin value had radii of curvature roughly equal to that of an ideal twin. However, below the ideal twin angle the radii increase rapidly with a greater variation in the values measured. This behaviour mirrors that of the twin boundaries shown in Supplementary Figure 5, where lower shears and weak compression allows the crystal’s basal planes to deviate from perfect atomic registry, which we observe as variations in the measured slip translation lengths.

The data in Supplementary Figure 6 were fitted with a simple asymptotic function:

$K(\theta)=\frac{A}{\theta-a\delta}$ (Supplementary Equation 2)

where *K*(θ) is the normalized radius of curvature, converted from the radius of curvature measured in HAADF STEM images using

$K(\theta)=\frac{r_{2}^{2}\left( N_{l}-1 \right)\left( 1-\alpha_{1}\alpha_{2} \right)e_{0}}{N_{l}\beta}$ (Supplementary Equation 3)

where *r_2_* is the radius of curvature, *N_l_* the thickness of the flake, *e_0_* the stacking fault energy, β the bending modulus and *α_1_* and *α_2_* are delamination coefficients (see Supplementary Note 3 for more details).

The fit gives $\delta$ = 31.9° ± 0.2° which corresponds to the twin onset angle. This is in good agreement with previous work carried out on MoS_2_, graphene and hBN.^14,15^ If a twin boundary is bent below this angle we expect the basal planes to slip over one another and form regular stacking with no twin boundary. The other fitting parameter *A* = 0.19 ± 0.02, is a function of the bending and adhesion properties of graphite.

**Supplementary Note 2.3: Formation Mechanism of Twin Boundaries**

In metals, a deformation twin boundary is formed when a subset of atoms in a crystal are moved distances no more than a single unit cell relative to the parent crystal lattice. This is achieved by bonds breaking and reforming in almost all directions. In this respect the twin boundaries in graphite are very different due to the presence of both very weak and very strong bonds. When a graphite crystal is deformed, the strong covalent bonds in the basal planes do not break. The van der Waals bonds between the sheets break instead and the sheets slide across one another, displacing atoms by many unit cell distances in the process. This process can be seen in Supplementary Movie 1 and Supplementary Figure 7, where a micromanipulator cleaves the top-most basal planes of graphite to form different bending phenomena under an SEM. This phenomenon has also be seen with in situ TEM for transition metal dichalcogenides.^15,16^ The mechanism is that, when the crystal is compressed in-plane and over a certain value, the layers bending, sliding over each other and also the formation of new surface associated with the delamination of layers, release the compression strain.

**Supplementary Note 2.4: Determining equivalent nanotube chirality of bent basal planes at high shears**

For small twin angles, thin crystals and the uppermost planes of thick crystals with small twin angles, we observe nanotube-like bending in the basal planes (see Figure 3 main text). The region of nanotube-like curvature is highlighted red respectively in Supplementary Figure 8a for hBN. Image filtering and segmentation allowed us to determine the radius of curvature of the individual basal planes. Discrete twins like those in Supplementary Figure 3 have a near constant radii of curvature, while the radii of curvature in nanotube-like boundaries decreases as the basal planes get closer to the apex of the bend. This is the same characteristic we would expect in a multiwalled nanotube: consecutive nanotubes with increasingly smaller radii on moving towards the core.

Using quantitative analysis of STEM HAADF images we can go further and not only determine this region to be the boundary equivalent of a multiwalled nanotube, but assign each curved basal plane an equivalent nanotube chirality (Supplementary Figure 8b). For each radius measured, **r_curve_**, we can calculate the equivalent circumference, *C*, of a theoretical full nanotube using:

$C=2\pi\mathbf{r}_{\mathbf{curve}}$ (Supplementary Equation 4)

It is also well known that the circumference of a carbon or hBN nanotube is related to the indices denoting chirality (**n**, **m**) by:

$C=a\sqrt{\mathbf{n}^{2}+\mathbf{m}^{2}+\mathbf{nm}}$ (Supplementary Equation 5)

where *a* is the lattice constant of the material. We have already determined the twin direction of the discrete twin boundaries to be ac from the slip translation vectors. From nanotube selection rules, this means that the chirality index **m** *= 0* and we can solve for chirality index **n** simply as:

$\mathbf{n}=\frac{2\pi\mathbf{r}_{\mathbf{curve}}}{a}$ (Supplementary Equation 6)

(For the other case of nanotubes running in the zz direction, i.e. a zz direction bend, the selection rule becomes ­**n** *=* **m**). Plotting the value of chirality index **n** for each basal plane (Supplementary Figure 8c) and fitting a line of best fit, we find the chirality index increases by 8**n** for each consecutive plane, with the smallest index **n** = 11. Using this we can assign each basal plane with nanotube-like curvature a chirality ([8*i* + 3], 0) where *i* is the plane index starting at 1. For example, the plane *i* = 3 has chirality (27, 0).

The basal plane at the very apex of the bend, coloured white in Supplementary Figure 8b, exhibits neither discrete twinning nor nanotube-like curvature. To explain this we can assign this plane, *i* = 0, a chirality of *(3,0)*. The equivalent radius of curvature is **r_curve_** = 0.12 nm. This radius is less than half of that predicted for the smallest carbon nanotube possible **r_curve_** = 0.3 nm. It is believed that the bond strain required to make a nanotube of this curvature is too great, and the lattice finds it more favourable to form in-plane defects to accommodate the shear strain. ^17,18^ We see this phenomenon in hBN and MoSe_2_, but not in graphite (see Supplementary Figure 19). This is perhaps due to the far greater stacking fault energy associated with hBN and MoSe_2_, whereas in graphite the basal planes can slip to avoid this defective bending of a single plane.

**Supplementary Note 3. Modelling the Twinning Process**

The boundary between the twinned and parent lattices of a 2D crystal involves the bending of the basal planes over a length of a few nm within the crystal. The HAADF STEM micrographs in main text Figure 2 and Supplementary Figure 3 show twin boundaries with narrow, discrete bending whereas main text Figures 3c and 3f shows regions of basal planes within the twin boundary with a mixture of local discrete bending and broader curved regions (further examples are provided in Supplementary Figure 19). The main constraint for formation of all these features is that on either side of the twin boundaries, the crystals must remain in registry (e.g. AB or AA’ stacking depending on the crystal structure) in order to minimise energy. The mechanics of these processes will now be analysed.

**Supplementary Note 3.1: Nanotube-like (NT) bending**

This process is shown schematically in Supplementary Figure 9. In this case the structure within the twin boundary is reminiscent of a segment of a multi-walled nanotube and consists of a basal planes in a series of concentric arcs subtending an angle, *θ*.

Assume that the interaction energy between each of the concentric layers is

$e_{1}=\alpha_{1}e_{0}$ (Supplementary Equation 7)

where $e_{0}$ is the van der Waals (vdW) interaction energy per unit area between adjacent layers in registry and $\alpha_{1}$ < 1.0 because of the reduced layer-layer interaction caused by the loss registry and a stacking fault.

The change of vdW interaction energy between layer $l$ and $l+1$ is then given by

$\Delta\epsilon_{vdW1}=\left( 1-\alpha_{1} \right)e_{0}\left[ r_{1}+\left( l-\frac{1}{2} \right)d \right]\theta b$ (Supplementary Equation 8)

where $\left[ r_{1}+\left( l-\frac{1}{2} \right)d \right]\theta$ is the average length of the two adjacent curved planes and $b$ is the width of the crystal.

If the number of layers of basal planes is $N_{l}$ then the total change of the vdW interaction energy is

$E_{vdW1}=\sum_{l=1}^{N_{l}-1} \Delta\epsilon_{vdW1}=\left( 1-\alpha_{1} \right)e_{0}\left[ (N_{l}-1)r_{1}+\frac{d}{2}\left( N_{l}-1 \right)^{2} \right]\theta b$ (Supplementary Equation 9)

This means that as *N_l_* increases, the vdW interaction energy increases approximately as *N_l_*^2^. The bending also leads to the deformation of the walls in the concentric region and the bending of the $l$-th layer leads to a bending energy of

$\Delta\epsilon_{bend1}= \beta\frac{1}{\left( r_{1}+\left( l-1 \right)d \right)^{2}}\left( r_{1}+\left( l-1 \right)d \right)\theta b=\frac{\beta\theta b}{r_{1}+\left( l-1 \right)d}$ (Supplementary Equation 10)

where $\beta$ represents the bending stiffness of the material.

The total bending energy is then

$E_{bend1}=\sum_{l=1}^{N_{l}} \Delta\epsilon_{bend1}\sim\frac{\beta\theta b}{d}\int_{\frac{r_{1}}{d}}^{\frac{r_{1}}{d}+N_{l}} \frac{1}{x}dx=\frac{\beta\theta b}{d}\ln\left( 1+\frac{(N_{l}-1)d}{r_{1}} \right)$ (Supplementary Equation 11)

This means that as *N_l_* increases, the bending energy increases approximately as ln*N_l_*. The unknown in such a model is $r_{1}$ and it can be determined by the minimization of the total energy of the system

$E_{tot1}=E_{vdW1}+E_{bend1}$ or

$E_{tot1}=\left( 1-\alpha_{1} \right)e_{0}\left[ (N_{l}-1)r_{1}+\frac{d}{2}\left( N_{l}-1 \right)^{2} \right]\theta b+$ $\frac{\beta\theta b}{d}\ln\left( 1+\frac{(N_{l}-1)d}{r_{1}} \right)$ (Supplementary Equation 12)

Differentiating this with respect to $r_{1}$ gives

$\frac{1}{r_{1}} -\frac{1}{r_{1}+N_{l}d}= \frac{1}{\beta}\left( 1-\alpha_{1} \right)e_{0}\left( N_{l}-1 \right)d$ (Supplementary Equation 13)

For the situation where ${N_{l}d\gg r}_{1}$, then

$r_{1}=\frac{\beta}{\left( N_{l}-1 \right)\left( 1-\alpha_{1} \right)e_{0}d}$ (Supplementary Equation 14)

**Supplementary Note 3.2: Discrete twinning (DT)**

For the case of discrete twinning (DT) each curved layer is bent discretely as shown in Supplementary Figure 10.

All the layers are deformed in exactly same way and the layer-to-layer distance in the bend increases from *d* to become

$d_{2}=d/\cos\left( \theta/2 \right)$ (Supplementary Equation 15)

We now introduce a coefficient $\alpha_{2}$ which describes the additional reduction in interlayer interaction energy due to the increased interlayer spacing within the discrete twin boundary. Combined with the decrease in atomic registry (described by the coefficient $\alpha_{1}$) this will produce a larger decrease in interaction energy for DT bending than was found for NT bending with the interaction energy for DT bending given by

$e_{2}=\alpha_{1}\alpha_{2}e_{0}$, ${(\alpha}_{2}<1.0$) (Supplementary Equation 16)

The change of vdW interaction for the formation of a twin in a crystal with *N_l_* layers is

$E_{vdW2}=(N_{l}-1)\left( 1-\alpha_{1}\alpha_{2} \right)e_{0}r_{2}\theta b$ (Supplementary Equation 17)

Consequently as *N_l_* increases, the increase in vdW interaction energy is proportional to *N_l_*. Similarly, the bend energy due to *N_l_* curved layers is

$E_{bend2}=N_{l}\frac{\beta\theta b}{r_{2}}$ (Supplementary Equation 18)

And hence as *N_l_* increases, the bending energy is also increases in proportional to *N_l_*. The unknown value of $r_{2}$ can be calculated by minimizing the total energy of the system

$E_{tot2}=E_{vdW2}+E_{bend2}$ or

$E_{tot2}=\left( N_{l}-1 \right)\left( 1-\alpha_{1}\alpha_{2} \right)e_{0}r_{2}\theta+N_{l}\frac{\beta\theta b}{r_{2}}$ (Supplementary Equation 19)

Differentiating this equation with respect to $r_{2}$ leads to

$r_{2}=\sqrt{\frac{N_{l}\beta}{\left( N_{l}-1 \right)\left( 1-\alpha_{1}\alpha_{2} \right)e_{0}}}$ (Supplementary Equation 20)

and in the case of *N_l_*  >>1, this becomes

$r_{2}=\sqrt{\frac{\beta}{\left( 1-\alpha_{1}\alpha_{2} \right)e_{0}}}$ (Supplementary Equation 21)

When $\theta$ is sufficiently large, there is a large increase in interlayer spacing ($d_{2}$ increases according to Supplementary Equation 16) and consequently the energy penalty for discrete twinning becomes very large (small $\alpha_{2}$). It then becomes more energetically favourable for the discrete twin to separate into two discrete twins each with smaller bend angles as shown schematically in Supplementary Figure 11.

**Supplementary Note 3.3: DFT Calculation of the input parameters for analysis of different bending modes**

The equations describing bending in the previous sections contain a number of unknown parameters ($e_{0}, e_{1}$ etc.) which are not measurable experimentally but which can be calculated using density functional theory (DFT). These DFT calculations were performed using the periodic plane-wave basis set code VASP 5.35^19^ and projector-augmented-wave potentials^20^. The exchange–correlation functional is described by the revised Perdew–Burke–Ernzerhof (PBE) exchange model with the empirical dispersion correction of Grimme (DFT-D2)^21^. A plane-wave cut-off of 400 eV was used for the graphene and hBN layers and a value of 280 eV was used for the MoSe_2_ layers. We used a Monkhorst–Pack *k*-point grid of 11×11×1 per unit, which ensured that the bond lengths and energies converged to within 0.001 nm and 1 meV, respectively.

The vdW interaction energy for perfect registry and ideal interlayer separation, $e_{0}$, can be found from our DFT calculations as the energy difference between two layers with a sufficiently large separation that they are non-interacting and that of a perfect bilayer in registry (upper plot Supplementary Figure 12). The reduction in energy caused by the weakening of this vdW interaction, ($e_{0}-e_{1})$ for NT bending and ($e_{0}-e_{2})$ for DT bending, can also be obtained directly from the DFT calculations. The reduced interaction energy for a bilayer in the NT case with ideal interlayer spacing, $e_{1}$, can be determined by considering the energy penalty for sliding the two layers past each other while maintaining the ideal interlayer spacing (lower plots Supplementary Figure 12). The average of the period is used such that

$e_{1}=\frac{\sum\Delta x_{i}\times E_{i}}{\sum\Delta x_{i}}=\alpha_{1}e_{0}$ (Supplementary Equation 22)

where $\Delta x_{i}$ is the change in sliding distance along the zz or ac directions. The reduced interaction energy for the bilayer in the discrete twinning case, $e_{2}$, can be determined by considering sliding the two layers past each other while allowing the interlayer separation to relax. Values of $e_{0}$, $(e_{0}-e_{1})$ and $(e_{0}-e_{2})$ calculated directly from the DFT calculations with the units of eV/area are given in Supplementary Table 1.

The bending stiffness of the planes of molecules, *β,* can also be determined from our DFT calculations. The total energy of a tube $E_{tube}$ and that of a monolayer $E_{ml}$ are first calculated. The length of the tube $b$ and its diameter $D$ can be measured from the relaxed structures. The bending stiffness $\beta$ can then be fitted from a set of tubes with different sizes according to the following equation:

$E_{tube}-E_{ml}=\beta\frac{\mathrm{area}}{\mathrm{radius}^{2}}=\beta\frac{\pi Db}{\left( \frac{D}{2} \right)^{2}}=\beta\frac{4\pi b}{D}$ (Supplementary Equation 23)

**Supplementary Note 3.4: Deformation Analysis – Radii of Curvature**

A suitable radius of curvature, $r$, for the bent planes in the twin should minimize the total energy of the system. For both nanotube-like bending and discrete twinning, the energy satisfies the relationships: $\Delta\epsilon_{vdW}\sim r$ and $\Delta\epsilon_{bend}\sim\frac{1}{r}$. Therefore as $r$ increases, $E_{vdW}$ increases, whereas $E_{bend}$ decreases. We can therefore find an optimal value of the radius of curvature for each bending mode (r_1_ for NT and r_2_ for DT) in a crystal with a given number of layers, *N_l_*, as shown in Supplementary Figure 13.

A comparison of the radii of curvature measured from the STEM micrographs and the values predicted by the DFT theory is given in Supplementary Figure 14 for NT and DT bending modes. It can be seen that there is quite good agreement for NT bending but the theory somewhat underestimates the radii of curvature for DT bending. No adjustment has been made in the parameters determined by DFT.

**Supplementary Note 3.5: Effect of the number of layers upon the twinning modes**

It is also useful consider how the energetics of the NT and DT twinning modes changes as the number of layers, *N_l_* increases. This is summarized in Supplementary Figure 15 for twinning with traces in the ac direction in graphite. It can be seen that the energy per unit width increases linearly with *N_l_* for DT twins but non-linearly (approximately as *N_l_*^2^ for higher *N_l_*) for NT bending. For a small number of layers NT bending therefore has the lowest energy whereas as at a critical value of *N_l_* , DT twinning has the lowest energy and therefore becomes more favourable. The dependence of the radius of curvature, *r*_1_ or *r*_2_ for the two modes is also plotted in Supplementary Figure 15 as a function of *N_l_*.

**Supplementary Note 3.6: Deformation at High Bend Angles**

As discussed previously when the bend angle, *ϕ*, becomes sufficiently large, a discrete twin is expected to split into more than one lower angle twin as shown in Supplementary Figure 11 and observed in Figure 3 in the main text. The variation of *α*_2_ with *ϕ*, calculated using DFT shows a sharp drop in *α*_2_ as *ϕ* increases (Supplementary Figure 16). Consequently, it will always be energetically favourable for a discrete twin with *ϕ* = 2*θ* to split into two discrete twins of *ϕ* = *θ.*

Figure 4 of the main text demonstrates that experimentally we find that as well as high angle twins splitting into more than one discrete twin, there is also always a region of nanotube-like bending at the core of the twin, which we describe as a mixed bending mode. In Supplementary Notw 3.5, it was shown that NT bending has lower energy when $N_{l}$ is smaller than $N_{c}$, otherwise discrete twining has lower energy and so is more stable. However, we now consider the energetics of hybrid mixtures of NT bending and DT bending to minimise the overall energy of the bend if $N_{l}$ is larger than a critical value $N_{0}$. This situation is visualised schematically in Supplementary Figure 17.

It is envisaged that the structure in the mixed mode is a combination of $N_{c}^{iii}$ layers of NT bending and $(N_{l}-N_{c}^{iii})$ layers of DT bending. Ignoring the interaction between the $N_{c}^{iii}$*th* and the $(N_{c}^{iii}+1)$*th* layers then the components of the total energy are as follows

$E_{vdW3}=\sum_{l=1}^{N_{c}^{iii}-1} \Delta\epsilon_{vdW1}+\sum_{N_{c}^{iii}}^{N_{l}-1} \Delta\epsilon_{vdW2}$ and $E_{bend3}=\sum_{l=1}^{N_{c}^{iii}} \Delta\epsilon_{bend1}+\sum_{N_{c}^{iii}}^{N_{l}} \Delta\epsilon_{bend2}$ (Supplementary Equation 24)

and the optimal values of $N_{c}^{iii}, r_{1}, r_{2}$ can be calculated for each value of $N_{l}$ from $\frac{\boldsymbol{\partial}(E_{vdW3}+E_{bend3})}{\boldsymbol{\partial}N_{c}^{iii} \boldsymbol{\partial}r_{1} \boldsymbol{\partial}r_{2}}=0$

The energetics of the situation is summarized in Supplementary Table 2. When $N_{l}<N_{0}$, the bending of each layer will follow NT bending. For $N_{l}>N_{0}$, layers with discrete twinning will be present, then $N_{c}^{iii}=N_{0}$

Hence to summarize $N_{c}^{iii}=\left\{ \begin{aligned} N_{l}, N_{l}<N_{0} \\ N_{0}, N_{l}\geq N_{0} \end{aligned} \right.$

The energetics of the different twinning modes for an ac twin in graphite with an angle *ϕ* = 84° are shown in Supplementary Figure 18. A single DT twin with *θ* = 84° will split preferentially into two DT twins with *θ* = 42°. It is interesting to note that the value of $N_{c}=30$ is the same as that for the case of *θ* = 42° shown in Supplementary Figure 15. This is because the NT bending energy is proportional to *θ* and so doubles for *θ* = 84°, and the DT energy is twice that of two single twins of *θ* = 42°.

It also follows that once the material is thick enough to form a double discrete twin, it can reduce its energy further by undergoing mixed-mode bending with NT bending in the layers below *N*_0_ (where for the situation shown in Supplementary Figure 18 *N*_0_ = 16 ) and with two discrete twins in the layers above *N*_0_.

**Supplementary Note 3.7: Stacking of Molecular Layers in Crystals of 2D materials**

Our results suggest that the stacking of the molecular layers in the 2D materials has a major influence upon the twinning mechanisms observed. The different types of stacking in the three materials considered in this work are shown in Supplementary Figure 20. Graphite prefers AB Bernal stacking whereas hBN and MoSe_2_ prefer AA’ stacking.

In all three cases, ac twinning involving shear in the zz-direction by one lattice translation vector, *a*, restores the packing and hence the lattice. However, the situation is different for zz twinning involving shear in the ac-direction because of the difference in packing and the different chemical nature of the atoms. For hBN and MoSe_2_ the interlayer bonding is restored by a shear of √3*a*. The situation in graphite, however, is more complicated because there are 3 equivalent sites in the lattice, as shown in Supplementary Figure 21. One graphene layer is therefore able to slide over another in the AC-direction to a series of intermediate positions in steps of *a/*√3 although AB stacking is only restored for a full shear of 3*a/*√3=√3*a*. The intermediate shears of *a/*√3 and 2*a/*√3 produce structures with different types of stacking within the twin. This is essentially ..AABBCC.. stacking, i.e. rhombohedral stacking with a stacking fault on every other plane. Freise and Kelly^1,2^ foresaw this problem and suggested that such a twin could accommodate atomic shuffles required to reproduce the Bravais lattice with Bernal stacking by a shear displacement of alternating (0001) basal planes by $\frac{1}{3}a[10\bar{1}0]$ and $\frac{1}{3}a[01\bar{1}0]$ as was shown in Supplementary Figure 2.

**Supplementary Note 4. Delamination in thick van der Waals’ crystals at high shear**

Supplementary Figures 22a, 22c, and 22d, shows that large cracks between basal planes can form from high shears about the ac direction in graphite, hBN and MoSe_2_. We hypothesise that this must be present only in thick crystals, as Supplementary Figure 22b shows that relatively thin graphite flakes (in this case approximately 30 basal planes) do not delaminate under the highest shear possible: a bend angle of 180°. Cracks form when groups of basal planes slip past one another by multiple translation lengths to form thin, delaminated sections with nanotube-like curvature. The thin sections comprise of only 2 – 4 basal planes in each material. We find this thickness is material and shear dependent, and also expect different behaviour for bending about the zz direction.

**Supplementary Note 5. Twinning and Mechanical Exfoliation**

Although there have been a considerable number of studies upon the bulk mechanical exfoliation of crystals of 2D materials by processes such as ultrasonic or shear exfoliation, the mechanisms are still not yet understood. The main features are summarised below:

- The exfoliation process reduces the number layers in the crystals.
- The lateral dimensions of the flakes as well as their thicknesses are also reduced.
- The fractured edges of the exfoliated flakes are normally parallel to armchair or zigzag directions^22,23^.
- A small amount of monolayers are produced but the majority of the material at the end of the exfoliation process is mainly few-layer flakes (~8 layer graphene in the case of the shear exfoliation of graphite^24^).

Paxton et al.^24^ suggested that the exfoliation process takes place like the sliding of a deck of cards by shear of one half of a crystal over the other but this only accounts for the reduction in crystal thickness and is unlikely since it would be difficult for a liquid to apply a sufficiently-large shear stress to a crystal for this to take place.

From the results of this present study we propose that twinning plays a pivotal role in the mechanical exfoliation of 2D crystals. Firstly, twinning has been shown to be the easiest and most common deformation mechanism that can be activated in crystals of 2D materials subjected to stress. Supplementary Figure 23 shows that both zz and ac twins are found in deformed crystals with traces along the zigzag and armchair directions respectively. Cracks are also seen in the crystal running along the same crystallographic directions. Although it is well-established that fracture is favoured along the armchair and zigzag directions^22,23^, it is likely that the high degree of localised deformation within the twins facilitates fracture along these directions.

Secondly, Supplementary Figure 23b shows that cracks form within high-angle twins at the transition point between nanotube-like and discrete twinning. Such cracks will act as nucleation points for delamination during ultrasonic or shear exfoliation. At high magnification (Supplementary Figure 23c) it can be seen that the segments comprise more than one layer (an average of approximately 6 in this case for MoSe_2_) which has been shown earlier to be controlled by the balance between nanotube bending and discrete twinning. This explains why bulk mechanical exfoliation processes do not generally produce monolayer. When the delamination occurs, it can be expected that the crest of the twinning fractures that eventually leads to the breaking of the top graphene flakes into two or more fragments. Specifically, when the twins meet others, the junction area has a crystallographic mismatch and high strain energy therefore it is very likely to fracture and delaminate, as can be found in Supplementary Figures 23d and 23e. At the crest of the junction of twins, the graphene flake is significantly twisted and the formation of cracks can be seen which leads to further fragmentation of the top flake. It can also occur in an alternative way that the whole top flake buckles and forms a hollow structure, the surface of which can be very weak and easy to fracture.

**Supplementary Note 6. Raman Spectra of Twins**

The structure of two twins crossing at 90° was examined using Raman spectroscopy in steps across two twins as shown in Supplementary Figure 24. It can be seen that the shape of the 2D band changes markedly across the zz twin, to a broadened band, but is unchanged across the ac twin.

The absence of the D band in the spectra for all three areas implies that the change in shape of the 2D band is not due to defects or crystal size change^25^. It is well established that the stacking of monolayers in few-layer graphene has a strong effect upon the shape and position of the 2D Raman band^26–28^. When graphite changes from Bernal (ABA) stacking to rhombohedral (ABC) stacking, the main change in the Raman spectrum would be an enhanced shoulder at the low wavenumber side of the 2D band^28–31^. This is in good agreement of the Raman results in Figure 5 main text in which the 2D band of the zz twin changes significantly from that of pristine graphite and ac twin. This allows us to use the intensity ratio of the two components of the 2D band as a straightforward way to determine the type of twin (as discussed in the main text).

However, there are also other factors that can cause changes to the shape of the 2D band and we have therefore considered the broader Raman spectrum (Supplementary Figure 25), comparing the Raman spectra for pristine graphite and on each of the two types of twin (ac and zz). It can be again seen that the Raman spectrum is the same for the pristine graphite and the ac twin; however it changes markedly on the zz twin. The M band around 1750 cm^-1^ is an out-of-plane transverse mode related to the AB stacking due to the interlayer coupling^32,33^. It is an asymmetric band for AB stacked graphite however splits when the stacking changes to ABC^33^. Another feature, the G* band (~ 2450 cm^-1^) also has a shape change for different stacking orders^34^. These M band and G* band are related to the ABA stacking regardless the number of layers, hence the shape change of 2D band in these twins is unlikely to be from delamination^26,31,33^. Considering, the behaviour of the M-band and G* band as well as the 2D band all our results imply that in the zz twin, there is some change of atomic stacking from ABA to ABC.

**Supplementary References**

1. Kelly, A. & Knowles, K. M. *Crystallography and crystal defects*. (Wiley, MA, 2012).

2. Freise, E. J. & Kelly, A. Twinning in Graphite. *Proc. R. Soc. Lond. Math. Phys. Eng. Sci.*, **264,** 269–276 (1961).

3. Young, R. J., Bloor, D., Batchelder, D. N. & Hubble, C. L. Deformation mechanisms in polymer crystals. *J. Mater. Sci.*, **13,** 62–71 (1978).

4. Young, R. J., Dulniak, R., Batchelder, D. N. & Bloor, D. Twinning in macroscopic polymer single crystals. *J. Polym. Sci. Polym. Phys. Ed.*, **17,** 1325–1339 (1979).

5. Thomas, J. M., Glenda Hughes, E. E. & Williams, B. R. Unusual Twinning in Graphite. *Nature*, **197,** 682–683 (1963).

6. Kennedy, A. J. Dislocations and Twinning in Graphite. *Proc. Phys. Soc.*, **75,** 607 (1960).

7. Haigh, S. J., Gholinia, A., Jalil, R., Romani, S., Britnell, L., Elias, D. C., Novoselov, K. S., Ponomarenko, L. A., Geim, A. K. & Gorbachev, R. Cross-sectional imaging of individual layers and buried interfaces of graphene-based heterostructures and superlattices. *Nat Mater*, **11,** 764–767 (2012).

8. Georgiou, T., Jalil, R., Belle, B. D., Britnell, L., Gorbachev, R. V., Morozov, S. V., Kim, Y. J., Gholinia, A., Haigh, S. J., Makarovsky, O., Eaves, L., Ponomarenko, L. A., Geim, A. K., Novoselov, K. S. & Mishchenko, A. Vertical field-effect transistor based on graphene-WS2 heterostructures for flexible and transparent electronics. *Nat. Nanotechnol.*, **8,** 100–103 (2013).

9. Peña, F. de la, iygr, Ben, Walls, M., Sarahan, M., Caron, J., Garmannslund, A., Ostasevicius, T., Petras, Mazzucco, S., Eljarrat, A., Burdet, P., Prestat, E., Taillon, J., Donval, G., Johnstone, D., Fauske, V. T., MacArthur, K. E., Nord, M., Furnival, T., Zagonel, L. F. & Aarholt, T. *hyperspy: HyperSpy 0.8.5*. (2016).

10. Hunter, J. D. Matplotlib: A 2D graphics environment. *Comput. Sci. Eng.*, **9,** 90–95 (2007).

11. van der Walt, S., Schönberger, J. L., Nunez-Iglesias, J., Boulogne, F., Warner, J. D., Yager, N., Gouillart, E. & Yu, T. scikit-image: image processing in Python. *PeerJ*, **2,** e453 (2014).

12. Walt, S. van der, Colbert, S. C. & Varoquaux, G. The NumPy Array: A Structure for Efficient Numerical Computation. *Comput. Sci. Eng.*, **13,** 22–30 (2011).

13. Schaffer, M., Schaffer, B. & Ramasse, Q. Sample preparation for atomic-resolution STEM at low voltages by FIB. *Ultramicroscopy*, **114,** 62–71 (2012).

14. Nikiforov, I., Tang, D.-M., Wei, X., Dumitricǎ, T. & Golberg, D. Nanoscale Bending of Multilayered Boron Nitride and Graphene Ribbons: Experiment and Objective Molecular Dynamics Calculations. *Phys. Rev. Lett.*, **109,** 025504 (2012).

15. Tang, D.-M., Kvashnin, D. G., Najmaei, S., Bando, Y., Kimoto, K., Koskinen, P., Ajayan, P. M., Yakobson, B. I., Sorokin, P. B., Lou, J. & Golberg, D. Nanomechanical cleavage of molybdenum disulphide atomic layers. *Nat. Commun.*, **5,** 3631 (2014).

16. Casillas, G., Santiago, U., Barrón, H., Alducin, D., Ponce, A. & José-Yacamán, M. Elasticity of MoS2 Sheets by Mechanical Deformation Observed by in Situ Electron Microscopy. *J. Phys. Chem. C*, **119,** 710–715 (2015).

17. Qin, L.-C., Zhao, X., Hirahara, K., Miyamoto, Y., Ando, Y. & Iijima, S. Materials science: The smallest carbon nanotube. *Nature*, **408,** 50–50 (2000).

18. Zhao, X., Liu, Y., Inoue, S., Suzuki, T., Jones, R. O. & Ando, Y. Smallest Carbon Nanotube Is 3 Angstrom in Diameter. *Phys. Rev. Lett.*, **92,** 125502 (2004).

19. Kresse, G. & Hafner, J. Ab initio molecular dynamics for liquid metals. *Phys. Rev. B*, **47,** 558–561 (1993).

20. Blöchl, P. E. Projector augmented-wave method. *Phys. Rev. B*, **50,** 17953–17979 (1994).

21. Grimme, S. Semiempirical GGA-type density functional constructed with a long-range dispersion correction. *J. Comput. Chem.*, **27,** 1787–1799 (2006).

22. Geim, A. K. & Novoselov, K. S. The rise of graphene. *Nat Mater*, **6,** 183–191 (2007).

23. Ciesielski, A. & Samorì, P. Graphene via sonication assisted liquid-phase exfoliation. *Chem. Soc. Rev.*, **43,** 381–398 (2013).

24. Paton, K. R., Varrla, E., Backes, C., Smith, R. J., Khan, U., O’Neill, A., Boland, C., Lotya, M., Istrate, O. M., King, P., Higgins, T., Barwich, S., May, P., Puczkarski, P., Ahmed, I., Moebius, M., Pettersson, H., Long, E., Coelho, J., O’Brien, S. E., McGuire, E. K., Sanchez, B. M., Duesberg, G. S., McEvoy, N., Pennycook, T. J., Downing, C., Crossley, A., Nicolosi, V. & Coleman, J. N. Scalable production of large quantities of defect-free few-layer graphene by shear exfoliation in liquids. *Nat. Mater.*, **13,** 624–630 (2014).

25. Cançado, L. G., Jorio, A., Ferreira, E. H. M., Stavale, F., Achete, C. A., Capaz, R. B., Moutinho, M. V. O., Lombardo, A., Kulmala, T. S. & Ferrari, A. C. Quantifying Defects in Graphene via Raman Spectroscopy at Different Excitation Energies. *Nano Lett.*, **11,** 3190–3196 (2011).

26. Nguyen, T. A., Lee, J.-U., Yoon, D. & Cheong, H. Excitation Energy Dependent Raman Signatures of ABA- and ABC-stacked Few-layer Graphene. *Sci. Rep.*, **4,** (2014).

27. Gong, L., Young, R. J., Kinloch, I. A., Haigh, S. J., Warner, J. H., Hinks, J. A., Xu, Z., Li, L., Ding, F., Riaz, I., Jalil, R. & Novoselov, K. S. Reversible Loss of Bernal Stacking during the Deformation of Few-Layer Graphene in Nanocomposites. *ACS Nano*, **7,** 7287–7294 (2013).

28. Lui, C. H., Li, Z., Chen, Z., Klimov, P. V., Brus, L. E. & Heinz, T. F. Imaging Stacking Order in Few-Layer Graphene. *Nano Lett.*, **11,** 164–169 (2011).

29. Zhang, W., Yan, J., Chen, C.-H., Lei, L., Kuo, J.-L., Shen, Z. & Li, L.-J. Molecular adsorption induces the transformation of rhombohedral- to Bernal-stacking order in trilayer graphene. *Nat. Commun.*, **4,** 2074 (2013).

30. Zhang, X., Han, W.-P., Qiao, X.-F., Tan, Q.-H., Wang, Y.-F., Zhang, J. & Tan, P.-H. Raman characterization of AB- and ABC-stacked few-layer graphene by interlayer shear modes. *Carbon*, **99,** 118–122 (2016).

31. Henni, Y., Ojeda Collado, H. P., Nogajewski, K., Molas, M. R., Usaj, G., Balseiro, C. A., Orlita, M., Potemski, M. & Faugeras, C. Rhombohedral Multilayer Graphene: A Magneto-Raman Scattering Study. *Nano Lett.*, **16,** 3710–3716 (2016).

32. Brar, V. W., Samsonidze, G. G., Dresselhaus, M. S., Dresselhaus, G., Saito, R., Swan, A. K., Ünlü, M. S., Goldberg, B. B., Souza Filho, A. G. & Jorio, A. Second-order harmonic and combination modes in graphite, single-wall carbon nanotube bundles, and isolated single-wall carbon nanotubes. *Phys. Rev. B*, **66,** 155418 (2002).

33. Cong, C., Yu, T., Saito, R., Dresselhaus, G. F. & Dresselhaus, M. S. Second-Order Overtone and Combination Raman Modes of Graphene Layers in the Range of 1690−2150 cm−1. *ACS Nano*, **5,** 1600–1605 (2011).

34. Cong, C., Yu, T., Sato, K., Shang, J., Saito, R., Dresselhaus, G. F. & Dresselhaus, M. S. Raman Characterization of ABA- and ABC-Stacked Trilayer Graphene. *ACS Nano*, **5,** 8760–8768 (2011).
