# Supplementary material for: Anomalous twin boundaries in two dimensional materials
Source: Nat Commun. 2018 Sep 5;9:3597. doi: 10.1038/s41467-018-06074-8 (PMC6125487; doi:10.1038/s41467-018-06074-8)
Supplement: Supplementary file 2 — Description of Additional Supplementary Files [file 41467_2018_6074_MOESM2_ESM.pdf]

**Description of Additional Supplementary Files**

File Name: Supplementary Movie 1
Description: Showing where a micromanipulator cleaves the top-most basal planes of graphite to form different bending phenomena observed in an SEM.
